# Role of oxidative balance score in staging and mortality risk of cardiovascular-kidney-metabolic syndrome: Insights from traditional and machine learning approaches

**DOI:** 10.1016/j.redox.2025.103588

**Published:** 2025-03-07

**Authors:** Yang Chen, Shuang Wu, Hongyu Liu, Ziyi Zhong, Tommaso Bucci, Yimeng Wang, Manlin Zhao, Yang Liu, Zhengkun Yang, Ying Gue, Garry McDowell, Bi Huang, Gregory Y.H. Lip

**Affiliations:** a Liverpool Centre for Cardiovascular Science at University of Liverpool, Liverpool John Moores University and Liverpool Heart and Chest Hospital, Liverpool, United Kingdom; b Department of Cardiovascular and Metabolic Medicine, Institute of Life Course and Medical Sciences, University of Liverpool, Liverpool, United Kingdom; c National Center for Cardiovascular Disease, Fuwai Hospital, Chinese Academy of Medical Sciences and Peking Union Medical College, Beijing, People's Republic of China; d National Clinical Research Center of Cardiovascular Diseases, National Center for Cardiovascular Disease, Fuwai Hospital, Chinese Academy of Medical Sciences and Peking Union Medical College, Beijing, People's Republic of China; e Department of Cardiovascular Medicine, The Second Affiliated Hospital, Jiangxi Medical College, Nanchang University, Nanchang, Jiangxi, People's Republic of China; f Department of Musculoskeletal Ageing and Science, Institute of Life Course and Medical Sciences, University of Liverpool, United Kingdom; g Department of Clinical Internal, Anaesthesiologic and Cardiovascular Sciences, Sapienza University of Rome, Rome, Italy; h Department of Cardiology, Beijing Anzhen Hospital, Capital Medical University, Engineering Research Center of Medical Devices for Cardiovascular Diseases, Ministry of Education, National Clinical Research Center for Cardiovascular Diseases, Beijing, People's Republic of China; i Department of Cardiology, Tianjin Medical University General Hospital, Tianjin, People's Republic of China; j School of Pharmacy and Biomolecular Sciences, Liverpool John Moores University, Liverpool, United Kingdom; k Department of Cardiology, The First Affiliated Hospital of Chongqing Medical University, Chongqing, People's Republic of China; l Danish Centre for Health Services Research, Department of Clinical Medicine, Aalborg University, Aalborg, DK-9220, Denmark; m Medical University of Bialystok, Bialystok, Poland

**Keywords:** Oxidative balance score, Cardiovascular-kidney-metabolic syndrome, Mortality, Oxidative stress, Risk stratification

## Abstract

**Objectives:**

To evaluate the roles of oxidative balance score (OBS) in staging and mortality risk of cardiovascular-kidney-metabolic syndrome (CKM).

**Methods:**

Data of this study were from the National Health and Nutrition Examination Survey 1999–2018. We performed cross-sectional analyses using multinomial logistic regression to investigate the relationship between OBS and CKM staging. Cox proportional hazards models were used to assess the impact of OBS on mortality outcomes in CKM patients. Additionally, mediation analyses were performed to explore whether OBS mediated the relationships between specific predictors (Life's Simple 7 score [LS7], systemic immune-inflammation index [SII], frailty score) and mortality outcomes. Then, machine learning models were developed to classify CKM stages 3/4 and predict all-cause mortality, with SHapley Additive exPlanations values used to interpret the contribution of OBS components.

**Results:**

21,609 participants were included (20,319 CKM, median [IQR] age: 52.0 [38.0–65.0] years, 54.3% male, median [IQR] follow-up: 9.4 [5.3–14.1] years). Lower OBS quartiles were associated with advanced CKM staging. Moreover, lower OBS quartiles were related to increased mortality risk, compared to Q4 of OBS (all-cause mortality: Q1: HR 1.31, 95% CI 1.18–1.46, Q2: HR 1.27, 95% CI 1.14–1.42, Q3: HR 1.18, 95% CI 1.06–1.32; cardiovascular mortality: Q1: HR 1.44, 95% CI 1.16–1.79, Q2: HR 1.39, 95% CI 1.11–1.74, Q3: HR 1.26, 95% CI 1.01–1.57; non-cardiovascular mortality, Q1: HR 1.27, 95% CI 1.12–1.44, Q2: HR 1.23, 95% CI 1.08–1.40, Q3: HR 1.16, 95% CI 1.02–1.31), with optimal risk stratification threshold for OBS was 22. Additionally, OBS mediated (ranging 4.25%–32.85 %) effects of SII, LS7, frailty scores on mortality outcomes. Moreover, light gradient boosting machine achieved the highest performance for predicting advanced CKM staging (area under curve: 0.905) and all-cause mortality (area under curve: 0.875). Cotinine increased risk, while magnesium, vitamin B6, physical activity were protective.

**Conclusions:**

This study highlights OBS as a risk stratification tool for CKM, emphasizing oxidative stress's role in CKM staging and mortality risk management.

## Highlights

• This study first evaluates the role of OBS in staging and mortality risk in CKM.
• Lower OBS was linked to advanced CKM stages and higher all-cause, cardiovascular, and non-cardiovascular mortality risks.
• The optimal OBS cutoff for identifying high-risk CKM patients was 22.
• OBS mediated the effects of systemic immune-inflammation index, Life's Simple 7, and frailty score on mortality outcomes.
• LightGBM excelled in CKM staging and mortality prediction, with cotinine as the strongest pro-oxidant risk factor.

## Graphical abstract

**Figure:**
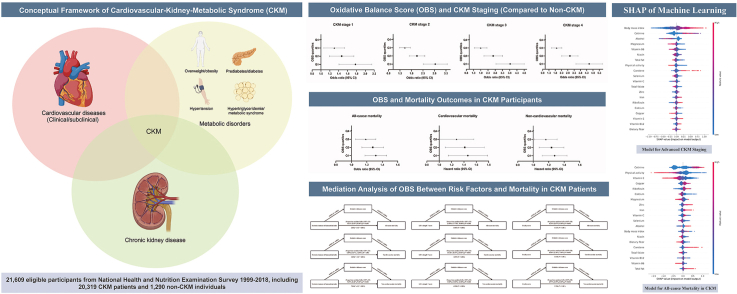

## Abbreviations list

AHA: American Heart Association
CKD: chronic kidney disease
CKM: cardiovascular-kidney-metabolism syndrome
CVD: cardiovascular disease
DM: diabetes mellitus
LS7: Life's Simple 7 Score
NHANES: National Health and Nutrition Examination Survey
OBS: oxidative balance score
PIR: poverty income ratio
ROS: reactive oxygen species
SII: systemic immune inflammation index

## 1 Introduction

The American Heart Association (AHA) has recently introduced the cardiovascular-kidney-metabolism syndrome (CKM), which is a systemic, progressive health disorder characterised by the coexistence of obesity, diabetes mellitus (DM), chronic kidney disease (CKD), and cardiovascular disease (CVD) [1]. The complex interactions and common pathological mechanisms among these conditions further exacerbate the burden of disease and the mortality risk. CKM affects a significant proportion (89.4 %) of the US adult population, with more than half of them at stage 2 or higher and no improvement between 2011 and 2020 [2]. A nationwide Italian primary care study reported a significant increase in hypertension, dyslipidaemia and obesity during the coronavirus disease 2019 pandemic, with a 1.7-fold sharp increase in pre-diabetes, as well as an increase in the prevalence of CKM with age, almost 50 % of the population having one or more complications of CKM [3].

The pathophysiology of CKM involves a complex spectrum of interplay mechanisms, where oxidative stress plays a key role. The hyperglycaemic state, impaired cardiac and renal function can lead to activation of the renin-angiotensin-aldosterone system on the one hand, and increases in reactive oxygen species (ROS), late glycosylation end products, and protein kinase C on the other hand, thus exacerbating oxidative stress, consequently exacerbating organ damage and dysfunction [[4], [5], [6]]. Therefore, comprehensive assessment of oxidative stress status is critically valuable for risk stratification of staging and mortality in CKM.

The oxidative balance score (OBS) is a holistic indicator that integrates 15 antioxidant and 5 pro-oxidant factors (16 dietary and 4 lifestyle factors) to assess an individual's oxidative stress status [7]. Typically, higher OBS reflects lower levels of oxidative stress and has been shown in studies to be strongly associated with the mortality risk from various chronic diseases, such as DM [7], CKD [8], and CVD [9]. However, no study has comprehensively examined the association between OBS and mortality risk in patients with CKM.

In this study, we focused on three main aspects: (1) examining the relationship between the OBS and the stages of CKM; (2) investigating the association between OBS and mortality risk in patients with CKM; and (3) exploring OBS as a potential mediator between various factors (e.g., Life's Simple 7 Score [LS7], frailty score) and mortality outcomes.

## 2 Methods

### 2.1 Data sources

Data of this study were obtained from the US nationally representative cohort, National Health and Nutrition Examination Survey (NHANES), which was approved by the National Center for Health Statistics Ethics Review Board. Since all data were anonymised, ethical review and informed consent for this study were waived. This study complies with the Strengthening the Reporting of Observational Studies in Epidemiology guideline (Supplementary Table S1).

### 2.2 Definitions of CKM and OBS

The definition of CKM in this study is shown in Supplementary Table S2. Here, the predicted 10-year CVD risk in subclinical CVD was calculated using the AHA PREVENT equations (Supplementary Table S3) [10], and renal function was categorised according to Kidney Disease: Improving Global Outcomes [11]. As defined by the AHA CKM definition, CKM was categorised into four stages (Supplementary Table S4) [1]. The OBS consists of 16 dietary nutrients and 4 lifestyle factors, the intake of each nutrient was calculated by averaging the two 24-h dietary recalls, incorporating contributions from diet and dietary supplements (Supplementary Table S5).

### 2.3 Study participants and sample size

Ten consecutive waves between 1999 and 2018 were included in this study, and of the 55,081 participants aged ≥20 years initially screened, 17,949 respondents with missing CKM-related information, 14,922 with missing OBS-related information, 385 with pregnancy, 27 without complete mortality follow-up, and 189 with extreme dietary intake were excluded, the final sample size was 21,609 (Supplementary Fig. S1). The extreme dietary intake was defined as less than 500 kcal/day or more than 5000 kcal/day for females, and less than 500 kcal/day or more than 8000 kcal/day for males [12].

### 2.4 Survival outcome

The survival outcomes were all-cause mortality, CVD mortality, and non-CVD mortality, which were sourced from the Centers for Disease Control and Prevention website (https://wwwn.cdc.gov/nchs/nhanes/Default.aspx) and were updated until December 31, 2019. The causes of mortality were identified using the tenth-vision International Statistical Classification of Diseases and Related Health Problems.

### 2.5 Definitions of covariates

Covariates extracted in this study included demographic information (age, sex, race and ethnicity, education level, poverty income ratio [PIR]), anthropometric measurements (weight, height, and waist circumference), lifestyle factors (physical activity, smoking status, and alcohol consumption), biomarkers (total cholesterol, highdensity lipoprotein cholesterol, estimated glomerular filtration rate, haemoglobin A1c), urine albumin to creatinine ratio, and C-reative protein), and other indicators (Life's Simple 7 score [LS7], systemic immune inflammation index [SII], and frailty score). Standard protocols were followed to measure weight, height, and waist circumference in the mobile examination center. Blood and urine samples were also collected there according to standard operating procedures, then processed, stored, and sent to the University of Minnesota in Minneapolis, MN for analysis. Body mass index (BMI) was determined by dividing weight (kg) by the height [2] (m^2^). Smoking status was categorised as follows: never smoker (<100 cigarettes smoked in lifetime), former smoker (≥100 cigarettes smoked but no smoking now), and current smoker (≥100 cigarettes smoked and currently smoking). Physical activity was categorised into three levels: “less than moderate,” “moderate,” and “vigorous,” based on intensity. Estimated glomerular filtration rate was derived from the Chronic Kidney Disease Epidemiology Collaboration Equation [13]. Urine albumin to creatinine ratio was calculated by urine albumin (ug/mL) dividing urine creatinine (mg/dL) multiple 100. SII was calculated using the formula: platelet count × neutrophil count/lymphocyte count [14]. The LS7 was defined based on AHA definitions, ranged from 0 to 14 points, with higher scores indicating better cardiovascular health (Supplementary Table S6 & Supplementary Table S7) [15]. The frailty score was evaluated through a comprehensive assessment method based on 49 frailty-related items (Supplementary Table S8) [16]. Race and ethnicity was self-reported by participants, including non-Hispanic White, non-Hispanic Black, Mexican American, Hispanic and others.

### 2.6 Statistical analysis

Some of the variables included in this study had varying proportions of missing values (Supplementary Table S9), all had less than 8 %, and we assumed that the missing data were Missing At Random. To handle missing values, we used the ‘miceforest’ package in Python, which employs multiple imputation of chained equations based on random forest model. The number of imputations was set to 10 iterations, and the imputed variables included education level, PIR, smoking status, physical activity, LS7, and SII, as these variables had missing values. Additionally, LS7 and SII were key variables in the mediation analysis, and the remaining covariates were included in the adjusted logistic regression and Cox proportional hazards models.

First, continuous variables were described by median and interquartile range (IQR) due to non-normal distribution and group comparisons were performed using the Kruskal-Wallis test. Categorical variables were described by counts and percentages, with group differences assessed using the Fisher's exact test. OBS quartile grouping was defined as quartile 1 (Q1): OBS<15; quartile 2 (Q2): 15≤ OBS <20; quartile 3 (Q3): 20≤ OBS <26; quartile 4 (Q4): OBS ≥26. Second, CKM staging was considered an ordinal variable, we used ordinal logistic regression to examine the relationship between OBS and CKM stages 0–4. However, the proportional odds assumption was violated, as indicated by the significant results of the test for parallel lines. Therefore, we performed multinomial logistic regression as an alternative to evaluate the associations between OBS and individual CKM stages, adjusted for age, sex, race and ethnicity, education level, PIR, smoking status, alcohol consumption, physical activity. Third, Kaplan-Meier survival curves were generated to illustrate cumulative mortality outcomes across OBS quartiles in CKM patients, and statistical differences were tested using the log-rank test. Fourth, restricted cubic spline (RCS) analyses were employed to visualise and explore the potential nonlinear associations between OBS with mortality outcomes in CKM patients. To determine the optimal number of knots, we calculated the Akaike Information Criterion (AIC) and Bayesian Information Criterion (BIC) for different knots (3–6) for model selection. Since our cohort is large-scale, to achieve a better balance between model complexity and generalisation capability, we preferentially refer to BIC for knots selection to reduce the risk of overfitting and improve the interpretability of the model. The results of AIC and BIC calculations and the finalised number of knots are detailed in Supplementary Table S10. Fifth, multivariable Cox proportional hazards models were constructed to estimate hazard ratios (HR) and 95 % confidence intervals (CI) for mortality outcomes across OBS quartiles, using the Q1 as the reference group. Models were adjusted for age, sex, race and ethnicity, education level, PIR, smoking status, alcohol consumption, physical activity. Additionally, the proportional hazards assumption was evaluated using Schoenfeld residuals, and the results confirmed that the assumption was satisfied. Sixth, subgroup analyses were performed to evaluate the influence of age (<65 vs. ≥65 years), sex, BMI (<30 vs. ≥30 kg/m^2^), and CKM staging group (1–2 vs. 3–4) on the associations between each OBS and mortality outcomes in CKM patients.

Additionally, we performed two sensitivity analyses. First, to avoid potential reverse causation, CKM patients who died within the first two years of follow-up were excluded, and the associations between each OBS and mortality outcomes were re-evaluated. Second, given the substantial impact of cancer on mortality outcomes, we examined the robustness of primary analysis after excluding CKM patients with a history of cancer.

Moreover, using the ‘surv_cutpoint’ function from the ‘survminer package’, we applied the maximally selected rank statistics method to identify the optimal risk stratification cut-off points for OBS on mortality outcomes [17].

Furthermore, to evaluate whether OBS serves as a mediator between specific predictors of mortality (LS7, SII, frailty score) and mortality outcomes, we performed mediation analysis using R with ‘mediation’ package. Bootstrapping with 1000 resamples was employed to estimate the 95 % CI of the mediation effect.

To further enhance the analysis, machine learning (ML) models—including light gradient boosting machine (LightGBM), random forest, logistic regression, support vector machine, and multi-layer perceptron—were developed to predict two outcomes: advanced CKM staging (CKM3/4) in whole patients and all-cause mortality in CKM patients. ML models were built in Python (version 3.11.4) with packages Scikit-learn (version 1.2.2) and lightgbm (version 3.3.5). The dataset was randomly split into a training set (70 %) and a testing set (30 %). The models incorporated 20 OBS components along with key clinical features (age and sex). To assess multicollinearity among the included variables, we calculated the variance inflation factor for all features. All variables had variance inflation factor values less than 8 (Supplementary Fig. S2 & Supplementary Fig. S3), indicating no significant multicollinearity and ensuring the stability and reliability of the model. Hyperparameter tuning was performed using five-fold cross-validation combined with randomised search and manual fine-tuning to optimise model performance in the training set. Model performance was evaluated in the testing set using receiver operating characteristic (ROC) curves, and metrics including the area under the curve (AUC), accuracy, specificity, precision, recall, F1-score, and G-mean. The best-performing model was selected based on these metrics, then we calculated Shapley Additive Explanations (SHAP) values to interpret and quantify the importance of OBS components in the best model for each of the two outcomes separately.

Descriptive analyses, Cox regression, and subgroup analyses were performed in SPSS Statistics (version 27, USA) following the official guidelines. Kaplan-Meier survival analyses, restricted cubic spline analyses, mediation analyses, and analyses for identifying optimal cut-off point were conducted in R (version 4.3.2, Austria). Multiple imputation and machine learning modelling process were implemented in Python (version 3.11.1, USA). The corresponding code for R and Python has been provided in the Supplementary Methods, where numerical parameters and file names have been replaced with placeholders (e.g., X) for clarity. Two-tailed *P* < 0.05 was regarded statistically significant.

## 3 Results

### 3.1 Baseline characteristics

21,609 eligible participants were included in this analysis (meadian [IQR] age: 52.0 [38.0–65.0] years, 54.3 % male, median [IQR] follow-up: 9.4 [5.3–14.1] years), including 20,319 CKM patients (median [IQR] age: 53.0 [40.0–66.0] years, 55.2 % male, median [IQR] follow-up: 9.3 [5.2–13.9] years) and 1290 non-CKM individuals (median [IQR] age: 32.0 [25.0–43.0] years, 37.5 % male, median [IQR] follow-up: 11.1 [6.9–16.1] years). Table 1 shows the baseline characteristics of CKM patients categorised by OBS quartiles. Participants in higher OBS quartiles had higher education levels, higher PIR, more vigorous physical activity, and lower proportion of smoking (all *P* < 0.001). Moreover, the proportion of individuals in advanced CKM staging (stages 3–4) decreased as OBS increased. Additionally, the baseline characteristics of participants categorised by CKM staging is presented in Supplementary Table S11.

**Table 1:** Baseline characteristics of all participants categorised by oxidative balance score quartiles.

Characteristics	All N = 20319	Q1 N = 4912	Q2 N = 4317	Q3 N = 5701	Q4 N = 5389	*P*
Age, years	53.0 (40.0, 66.0)	54.0 (40.0, 67.0)	54.0 (40.0, 66.0)	52.0 (39.0, 65.0)	53.0 (39.0, 65.0)	0.002
Age groups, n (%)						<0.001
Age ≤40 years	5435 (26.7)	1294 (26.3)	1132 (26.2)	1547 (27.1)	1462 (27.1)	
40< Age ≤50 years	3672 (18.1)	841 (17.1)	735 (17.0)	1091 (19.1)	1005 (18.6)	
50< Age ≤65 years	5989 (29.5)	1411 (28.7)	1296 (30.0)	1662 (29.2)	1620 (30.1)	
Age >65 years	5223 (25.7)	1366 (27.8)	1154 (26.7)	1401 (24.6)	1302 (24.2)	
Male, n (%)	11224 (55.2)	2856 (58.1)	2433 (56.4)	3067 (53.8)	2868 (53.2)	<0.001
Ethnicity, n (%)						<0.001
Non-Hispanic White	9960 (49.0)	2133 (43.4)	2043 (47.3)	2872 (50.4)	2912 (54.0)	
Non-Hispanic Black	4108 (20.2)	1394 (28.4)	957 (22.2)	1014 (17.8)	743 (13.8)	
Mexican American	3123 (15.4)	736 (15.0)	682 (15.8)	886 (15.5)	819 (15.2)	
Hispanic and Other	3128 (15.4)	649 (13.2)	635 (14.7)	929 (16.3)	915 (17.0)	
Body mass index, kg/m^2^	28.6 (25.3, 32.8)	29.6 (26.1, 33.8)	29.0 (25.8, 33.0)	28.6 (25.3, 32.9)	27.5 (24.4, 31.5)	<0.001
Waist circumference, cm	100.0 (90.9, 110.1)	102.5 (93.4, 112.6)	101.1 (92.2, 110.9)	99.7 (90.8, 110.0)	97.0 (88.6, 107.0)	<0.001
Education, n (%)						<0.001
Less than high school	1899 (9.3)	658 (13.4)	471 (10.9)	458 (8.0)	312 (5.8)	
High school or equivalent	7457 (36.7)	2178 (44.3)	1670 (38.7)	2036 (35.7)	1573 (21.1)	
College or above	10963 (54.0 %)	2076 (42.3)	2176 (50.4)	3207 (56.3)	3504 (65.0)	
Poverty income ratio	2.5 (1.2, 4.4)	1.93 (1.06, 3.54)	2.27 (1.21, 4.09)	2.64 (1.32, 4.59)	3.04 (1.50, 5.00)	<0.001
Poverty income ratio, n (%)						<0.001
<1	3456 (17.0)	1103 (22.5)	781 (18.1)	864 (15.2)	708 (13.1)	
1-3	8330 (41.0)	2236 (45.5)	1849 (42.8)	2292 (40.2)	1953 (36.2)	
≥3	8533 (42.0)	1573 (32.0)	1687 (39.1)	2545 (44.6)	2728 (50.6)	
Physical activity, n (%)						<0.001
Less than moderate	9515 (46.8)	2435 (49.6)	2038 (47.2)	2650 (46.5)	2392 (44.4)	
Moderate	6398 (31.5)	1524 (31.0)	1342 (31.1)	1830 (32.1)	1702 (31.6)	
Vigorous	4406 (21.7)	953 (19.4)	937 (21.7)	1221 (21.7)	1295 (24.0)	
Smoking status, n (%)						<0.001
Never smoker	10542 (51.9)	2148 (43.7)	2200 (51.0)	2980 (52.3)	3214 (59.6)	
Former smoker	5857 (28.8)	1333 (27.1)	1231 (28.5)	1692 (29.7)	1601 (29.7)	
Current smoker	3920 (19.3)	1431 (29.1)	886 (20.5)	1029 (18.0)	574 (10.7)	
Alcohol consumption, n (%)						<0.001
Non-drinker	12459 (61.3)	3021 (61.5)	2665 (61.7)	3420 (60.0)	3353 (62.2)	
Mild to moderate	4979 (24.5)	1112 (22.6)	1024 (23.7)	1442 (25.3)	1401 (26.0)	
Heavy	2881 (14.2)	779 (15.9)	628 (14.5)	839 (14.7)	635 (11.8)	
SBP, mmHg	126.0 (115.0, 138.0)	128.0 (117.0, 141.0)	126.0 (116.0, 138.0)	125.0 (115.0, 137.0)	124.0 (114.0, 136.0)	<0.001

DBP, mmHg	73.0 (65.0, 81.0)	74.0 (65.0, 82.0)	73.0 (65.0, 81.0)	73.0 (65.0, 81.0)	73.0 (65.0, 81.0)	0.256
Cancer, n (%)	2193 (10.8)	503 (10.2)	459 (10.6)	596 (10.5)	635 (11.8)	0.049
CKM stage, n (%)						<0.001
Stage 1	2506 (12.3)	438 (8.9)	462 (10.7)	767 (13.5)	839 (15.6)	
Stage 2	13478 (66.3)	3200 (65.1)	2868 (66.4)	3795 (66.6)	3615 (67.1)	
Stage 3	2011 (9.9)	558 (11.4)	461 (10.7)	535 (9.4)	457 (8.5)	
Stage 4	2324 (11.4)	716 (14.6)	526 (12.2)	604 (10.6)	478 (8.9)	
Laboratory indicators						
Hemoglobin A1c, %	5.5 (5.3, 5.9)	5.6 (5.3, 6.0)	5.6 (5.3, 6.0)	5.5 (5.3, 5.9)	5.5 (5.2, 5.8)	<0.001
Total Cholesterol, mg/dL	196.0 (170.0, 224.0)	196.0 (170.0, 225.0)	196.0 (169.0, 226.0)	196.0 (170.0, 224.0)	195.0 (170.0, 223.0)	0.495
HDL-C, mg/dL	50.0 (41.0, 61.0)	48.0 (40.0, 59.0)	49.0 (41.0, 59.0)	50.0 (41.0, 61.0)	52.0 (43.0, 64.0)	<0.001
eGFR, ml/min/1.73m^2^	91.8 (75.8, 106.4)	90.6 (73.7, 106.3)	91.4 (75.0, 106.5)	92.1 (76.1, 106.3)	92.5 (77.6, 106.5)	<0.001
UACR, mg/g	7.2 (4.5, 14.8)	7.9 (4.8, 18.2)	7.4 (4.5, 15.9)	6.9 (4.4, 13.6)	6.7 (4.4, 13.2)	<0.001
Multidimensional score						
SII	471.5 (337.9, 662.9)	480.0 (341.1, 688.0)	477.3 (339.3, 665.9)	468.3 (338.0, 658.0)	464.3 (333.1, 643.8)	0.002
Life's Simple 7 score	8.0 (7.0, 9.0)	7.0 (6.0, 8.0)	8.0 (6.0, 9.0)	8.0 (7.0, 9.0)	9.0 (7.0, 10.0)	<0.001
Frailty score × 10	1.3 (0.8, 1.9)	1.4 (0.9, 2.1)	1.3 (0.8, 2.0)	1.3 (0.8, 1.9)	1.2 (0.8, 1.7)	<0.001

Note: Q1: OBS<15; Q2: 15≤ OBS <20; Q3: 20≤ OBS <26; Q4: OBS ≥26.

P values from the Kruskal-Wallis test and the Fisher's exact test.

Abbreviations: CKM, cardiovascular-kidney-metabolic syndrome; DBP, diastolic blood pressure; eGFR, estimated glomerular filtration rate; HDL-C, high-density lipoprotein cholesterol; SBP, systolic blood pressure; SII, systemic immune-inflammation index; UACR, urinary albumin to creatinine ratio.

### 3.2 Distribution of mortality outcomes by OBS quartiles and CKM staging

As shown in Supplementary Fig. S4, all-cause mortality, cardiovascular mortality, and non-cardiovascular mortality decrease progressively across OBS quartiles (all *P* < 0.001). Specifically, all-cause mortality was highest in Q1 of OBS [20.3 %] and lowest in Q4 [10.8 %]. Similarly, cardiovascular mortality decreased from 5.3 % in Q1 to 2.5 % in Q4, while non-cardiovascular mortality declined from 15.0 % to 8.3 % across quartiles. Additionally, all-cause mortality was lowest in the non-CKM group (2.2 %) and significantly higher in CKM stages 3 and 4, at 48.1 % and 37.5 %, respectively. A similar trend was observed for cardiovascular and non-cardiovascular mortality.

### 3.3 Relationship between OBS and CKM staging

**Fig. 1:**
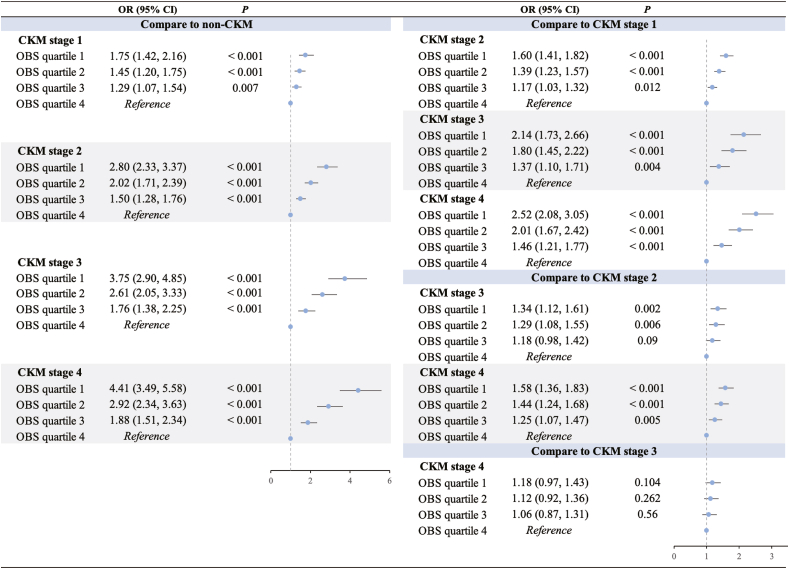
**Association Between OBS Quartiles and CKM Staging (Stages 0**–**4).** Q1: OBS<15; Q2: 15≤ OBS <20; Q3: 20≤ OBS <26; Q4: OBS ≥26. P values from multinomial logistic regression models adjusted for age, sex, race and ethnicity, education level, poverty income ratio, smoking status, alcohol consumption, physical activity. CI, confidence interval; CKM, cardiovascular-kidney-metabolic syndrome; OBS, oxidative balance score; OR, odds ratio.

Compared with Q4 of OBS, lower OBS quartiles were significantly associated with a higher likelihood of progressing from non-CKM to more advanced CKM staging (Fig. 1). For example, in Q1 of OBS, the odds ratios (OR) for progressing to CKM stages 1, 2, 3, and 4, compared to the non-CKM group, were 1.75 (95 % CI: 1.42–2.16), 2.80 (95 % CI: 2.33–3.37), 3.75 (95 % CI: 2.90–4.85), and 4.41 (95 % CI: 3.49–5.58), respectively (all *P* < 0.001). A similar trend was observed when CKM stage 1/2 was used as the reference group. However, when comparing CKM stages 3 and 4, the associations were attenuated and no longer statistically significant across most OBS quartiles.

### 3.4 Associations between OBS and mortality outcomes in CKM patients

**Fig. 2:**
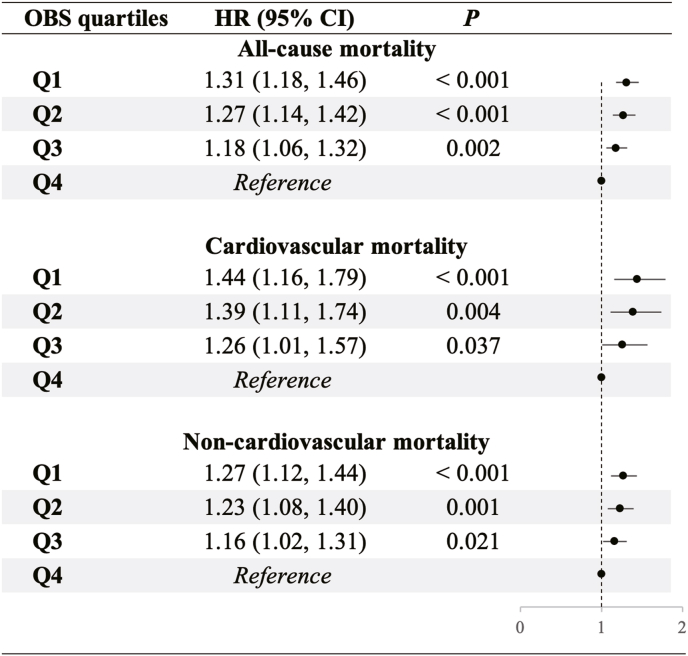
**Associations Between OBS and Mortality Outcomes in CKM Patients.** P values from multivariable Cox proportional hazards models adjusted for age, sex, race and ethnicity, education level, poverty income ratio, smoking status, alcohol consumption, physical activity. CI, confidence interval; CKM, cardiovascular-kidney-metabolic syndrome; HR, hazard ratio; OBS, oxidative balance score.

The KM survival curves (Supplementary Fig. S5) revealed that for all mortality outcomes, the Q1 of OBS group had the lowest probability of survival, whereas the Q4 of OBS group had the highest probability of survival, and all were statistically significant by Log-rank test (all *P* < 0.001). A significant negative linear relationship was found between OBS and all mortality outcomes (all *P-overall* < 0.001), but no non-linear relationship was found (Supplementary Fig. S6). Additionally, Fig. 2 shows that lower quartiles of OBS (Q1, Q2, Q3) were significantly associated with an increased risk of all-cause, cardiovascular and non-cardiovascular mortality compared to Q4 of OBS (all-cause mortality: Q1: HR 1.31, 95 % CI 1.18–1.46, Q2: HR 1.27, 95 % CI 1.14–1.42, Q3: HR 1.18, 95 % CI 1.06–1.32. Cardiovascular disease mortality: Q1: HR 1.44, 95 % CI 1.16–1.79, Q2: HR 1.39, 95 % CI 1.11–1.74, Q3: HR 1.26, 95 % CI 1.01–1.57. Non-cardiovascular disease mortality, Q1: HR 1.27, 95 % CI 1.12–1.44, Q2: HR 1.23, 95 % CI 1.08–1.40, Q3: HR 1.16, 95 % CI 1.02–1.31).

### 3.5 Subgroup analysis

The results of subgroup analysis suggested a significant interaction of age in the relationship between OBS and cardiovascular mortality (*P-interaction* = 0.003), with the remainder showing no significant interaction (Supplementary Table S12). Additional RCS analyses showed a significant negative association between OBS and cardiovascular mortality in those <65 years of age, whereas in those ≥65 years of age, the trend of negative association between OBS and the risk of cardiovascular mortality was more moderate, but a trend toward a decreasing risk was still present (Supplementary Fig. S7).

### 3.6 Sensitivity analysis

In sensitivity analyses, after excluding CKM patients who died within the first two-year follow-up and those with a history of cancer, and the association between each OBS and mortality outcome remained significant (Supplementary Table S13 and Supplementary Table S14).

### 3.7 Optimal risk stratification cut-off points for OBS on mortality outcomes in CKM patients

For all-cause, cardiovascular, and non-cardiovascular mortality outcomes, the optimal risk stratification cut-off point for OBS was 22 in all cases (Supplementary Fig. S8). According to Supplementary Table S15, each mortality outcome risk was notably elevated in patients with OBS ≥22 compared to those with OBS <22.

### 3.8 Mediation analysis

**Fig. 3:**
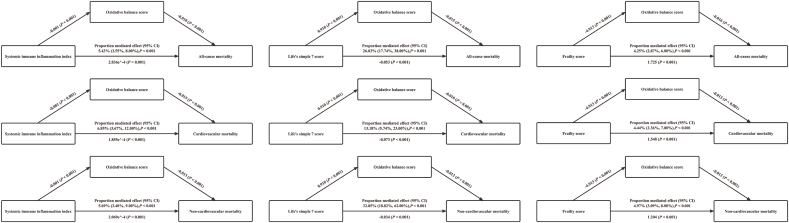
**Mediation Analysis of OBS in the Associations Between SII, LS7, Frailty Scores, and Mortality Outcomes in CKM Patients.** P values from the bootstrap sampling distribution or the normality assumption for statistical testing. CI, confidence interval; CKM, cardiovascular-kidney-metabolic syndrome; OBS, oxidative balance score.

Fig. 3 illustrates that OBS significantly mediated the associations of SII, LS7, and frailty scores with all-cause, cardiovascular, and noncardiovascular mortality outcomes (all *P* < 0.001). The proportions of effects mediated by OBS through the SII were 5.43 % (all-cause mortality), 6.85 % (cardiovascular mortality) and 5.69 % (non-cardiovascular mortality), respectively; the proportions of effects mediated by OBS through the LS7 were 26.03 %, 13.18 % and 32.85 %, respectively; and the proportions of effects mediated by OBS through the frailty score were 4.25 %, 4.44 % and 4.97 %, respectively.

### 3.9 Machine learning analysis

**Fig. 4:**
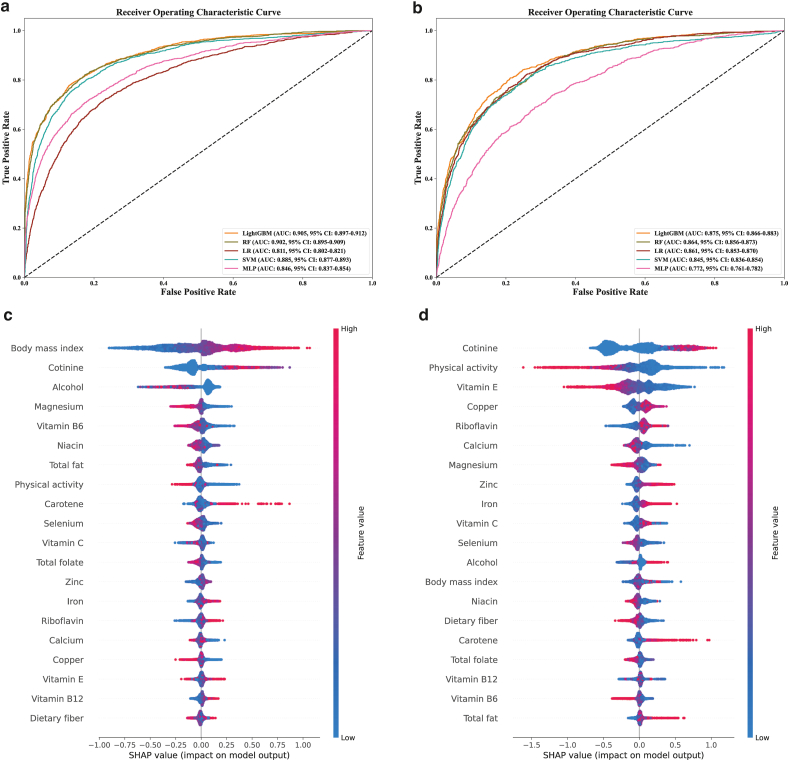
**ROC curves and SHAP-based feature importance of machine learning models in predicting advanced CKM staging and all-cause mortality.** (a) ROC curves for model in predicting advanced CKM staging, (b) ROC curves for model in predicting all-cause mortality in CKM patients, (c) SHAP summary plot for model in predicting advanced CKM staging, (d) SHAP summary plot for model in predicting all-cause mortality in CKM patients. AUC, area under the curve; CKM, cardiovascular-kidney-metabolic syndrome; LightGBM, light gradient boosting machine; LR, logistic regression; MLP, multi-layer perceptron; RF, random forest; ROC, receiver operating characteristic; SHAP, SHapley Additive exPlanations; SVM, support vector machine.

After inputting the OBS components and clinical characteristics into the five ML models, the best combinations of hyperparameters for the prediction models for predicting advanced CKM staging and all-cause mortality were determined after 5-fold cross-validation and manual fine-tuning, respectively (Supplementary Table S16 and Supplementary Table S17). For advanced CKM staging, ROC curves (Fig. 4a) and other metrics (Supplementary Table S18) were evaluated for all ML models in the testing set. LightGBM was considered to be the best model as it had the highest AUC (0.905), F1-score (0.668) and G-mean (0.677). For all-cause mortality in CKM, LightGBM was also the best performer with the highest AUC (0.875), F1-score (0.556) and G-mean (0.576) (Fig. 4b & Supplementary Table S19).

We calculated and ranked the corresponding SHAP values for each OBS component in the testing set for each of the two LightGBM models separately (Fig. 4c & d). In the LightGBM model for predicting advanced CKM staging, BMI and cotinine, both prooxidant factors, were the strongest predictors, indicating that higher BMI and tobacco exposure significantly increase the risk of CKM progression. Interestingly, higher alcohol intake was associated with a lower risk of CKM progression, though this inverse relationship should be interpreted cautiously due to potential confounding or the effects of moderate consumption. In contrast, the antioxidant components magnesium and vitamin B6 showed potential protective effects against CKM progression. In the LightGBM model for predicting all-cause mortality in CKM, cotinine was the most influential predictor, further highlighting the detrimental impact of tobacco exposure on survival among CKM patients. Conversely, higher physical activity, an antioxidant factor, was associated with a reduced risk of all-cause mortality. Moreover, increased levels of the antioxidants (copper and riboflavin) were linked to a higher risk of mortality, suggesting that their effects may be context-dependent or influenced by underlying health conditions. Additionally, elevated vitamin E intake, another antioxidant, was associated with a protective effect.

## 4 Discussion

This study explored the associations between the OBS, CKM staging, and mortality outcomes among CKM patients, while evaluating its mediating role in pathways of key factors and mortality outcomes. RCS analysis demonstrated a significant negative linear relationship between OBS and mortality outcomes. Higher OBS quartiles were linked to better baseline characteristics and lower risks of all-cause, cardiovascular, and non-cardiovascular mortality. Lower OBS was significantly associated with greater odds of progressing to advanced CKM staging. The optimal OBS cut-off for mortality stratification was identified as 22. Mediation analysis showed that OBS significantly mediated the relationships between SII, LS7, frailty score with mortality outcomes. Additionally, the LightGBM models demonstrated excellent performance in predicting both advanced CKM staging and all-cause mortality, with an average AUC of 0.905 and 0.875, respectively. SHAP analysis was used to interpret the model, highlighting the most influential features. For advanced CKM staging, BMI, cotinine, alcohol intake, magnesium, and vitamin B6 were identified as the top predictors. In contrast, for all-cause mortality, cotinine, physical activity, vitamin E, copper, and riboflavin were the most impactful factors. These results highlight the potential roles of maintaining an antioxidant-based diet and lifestyle in mitigating CKM staging and mortality risk management.

The association between OBS and the CKM staging and mortality risk can be interpreted through multifaceted biological mechanisms. Elevated levels of oxidative stress are associated with an activated inflammatory response, and sustained activation of inflammation leads to vascular damage and deterioration of organ function [18,19], which is reflected in the mediating effects associated with SII. Additionally, oxidative stress disrupts vascular endothelial homeostasis by increasing ROS production components, exacerbates arterial stiffness, and further promotes the progression of cardiovascular or kidney disease [20,21]. Moreover, oxidative stress induces insulin resistance, lipid metabolism disorders and glucose metabolism abnormalities [22], exacerbating the progression of CKM as well as increasing the mortality risk. OBS, as a comprehensive measure of oxidative homeostasis, integrates pro-oxidant and antioxidant factors, reflecting the overall burden of oxidative stress on the body in a more holistic way than a single marker of oxidative stress. Based on the mediation analyses in our study, this comprehensive assessment metric can reveal the global impact of oxidative stress in pathological status such as inflammation, metabolic imbalance, and frailty, further elucidating its critical role in the staging of CKM and mortality risk.

Our work is the first to validate the negative association between OBS and mortality risk in the CKM population, which supplements and expands the evidence from existing literature. Our results are similar to previous studies on the relationship between oxidative stress and the occurrence or prognosis of chronic diseases. Son et al. reported that higher OBS was significantly associated with a lower incidence of new-onset CKD (lowest tertile as reference; highest tertile for male: HR 0.70, 95 % CI 0.51–0.95; highest tertile for female: HR 0.73, 95 % CI 0.55–0.96) [23]. Kong et al. demonstrated that OBS was significantly negatively correlated with the prevalence of dyslipidaemia (Q1 as reference; Q2: OR 0.86, 95 % CI 0.77–0.97; Q3: OR 0.80, 95 % CI 0.72–0.91; Q4 OR 0.63, 95 % CI 0.56–0.70), and that continious OBS was also negatively associated with all-cause (HR 0.98, 95 % CI 0.98–0.99) and cardiovascualr mortality (HR 0.98, 95 % CI 0.97–0.99) in dyslipidaemia patients [24]. Additionally, Li et al. indicated that OBS was negatively linked to the prevalence of metabolic syndrome and its assessment components (Q1 as reference; Q4 for metabolic syndrome: OR 0.55, 95 % CI 0.47–0.64; Q4 for abdominal obesity: OR 0.61, 95 % CI 0.54–0.69; Q4 for hypertension: OR 0.69, 95 % CI 0.58–0.83) [25]. Notably, our study extends this evidence to the emerging concept of CKM, emphasizing the significance of OBS in this high-risk population.

Our study further highlights the clinical significance of OBS in mortality risk stratification of CKM patients. Specifically, we identified an optimal risk stratification threshold for OBS of 22, which is effective in identifying at-risk CKM populations and provides a valuable basis for developing targeted intervention strategies. Several studies have shown that different dietary patterns and antioxidant supplements can be effective in reducing levels of oxidative stress and maintaining metabolic homeostasis. For example, Yubero-Serrano and colleagues reported that patients who received the Mediterranean dietary intervention showed a significantly lower proportion of intracellular ROS-positive cells compared with the low-fat diet group, with a between-group difference of 11.1 % (95 % CI: 2.5 to 19.6) [26]. Thushara et al. found that crocin (100 μg/mL) effectively inhibited ROS generation in platelet-rich plasma (100.0 %) and washed platelets (99.0 %) [27]. Additionally, de Meirelles et al. demonstrated a significant increase in platelet superoxide dismutase and catalase activity in patients who received 6 months of chronic exercise supervision (30 min of moderate-intensity treadmill exercise, resistance and stretching) compared to control patients who did not receive this exercise (superoxide dismutase activity: 24,638 ± 3375 vs. 42,998 ± 6371 U/mg protein; catalase activity: 0.14 ± 0.01 vs. 0.16 ± 0.01 U/mg protein), suggesting that exercise training of appropriate intensity improves oxidative stress status [28]. These findings highlight the potential benefits of applying targeted dietary, supplement-based, and exercise interventions to high-risk CKM populations with OBS <22, aiming to reduce oxidative stress and improve clinical outcomes.

To the best of our knowledge, this analysis is the first to develop and validate ML models for predicting advanced staging and all-cause mortality risk in CKM. By integrating simple clinical features with OBS components, especially using the LightGBM model, the model performed well in CKM staging and mortality risk prediction, providing a new tool for risk stratification and management of CKM patients. The findings reveal the key role of antioxidants and pro-oxidants in CKM progression and mortality risk. Cotinine (a marker of smoking exposure) was one of the strongest predictors of advanced staging and mortality risk in CKM, highlighting the negative impact of smoking on the body's metabolic status [29]. BMI, a pro-oxidant, also significantly increased the risk of CKM progression, highlighting the need for interventions targeting poor lifestyles such as obesity and smoking. Notably, alcohol intake was associated with a lower risk of CKM staging, a result that may be related to the potential metabolic effects of moderate alcohol consumption or may be influenced by confounding factors. Ding et al. reported that that the lowest risk of mortality and recurrent cardiovascular events in individuals with CVD might be associated with lower levels of alcohol consumption [30]. Therefore, this inverse relationship needs to be interpreted with caution and further studies are needed to clarify the mechanism. In addition, some antioxidant components showed protective effects, such as higher magnesium and vitamin B6 intake were significantly associated with a lower risk of CKM progression, and higher physical activity and Vitamin E intake were also associated with a lower risk of death in CKM patients. However, elevated copper was associated with an increased risk of death, suggesting that certain antioxidants may be dose-dependent or associated with underlying metabolic abnormalities and that excessive intake may lead to toxicity due to copper accumulation. Li et al. demonstrated that excessive copper intake was related to an increased risk of mortality and CVD incidence among the general adult population in Asia [31]. These results emphasise the importance of maintaining oxidative homeostasis in the management of CKM. Future studies need to further explore the interaction mechanisms between antioxidants and pro-oxidants, clarify the dose effect, and develop more precise and individualised intervention strategies for CKM patients.

### 4.1 Limitations

This study has the following limitations. First, this was an observational study, which makes it difficult to determine the causal relationships between OBS and mortality risk, and prospective interventional studies are needed. Second, the calculation of OBS relied on dietary and lifestyle data, which may be subject to information bias and measurement error, especially when relying on retrospective self-reporting. Third, the sample was derived from the CKM population in the NHANES, and there may have been misclassification of the CKM staging due to some missing information and limitations of self-reported data, which may have affected our findings. Fourth, although we adjusted for various potential confounders, the effect of residual confounding on the results cannot be avoided. Fifth, the relationship between OBS and CKM staging was based on cross-sectional analyses, and time series and causality could not be determined; therefore, prospective studies are required. Sixth, the sample was primarily derived from the CKM population, and extrapolation of the results may be limited, pending validation in other populations. Seventh, the role of oxidative stress in cardiology remains controversial, and in light of this, our findings should be interpreted with caution, especially since OBS is not a direct measure of oxidative stress. Further prospective studies and mechanistic studies are needed to clarify the causal role of oxidative stress in CKM-related mortality and to validate the utility of OBS as a predictor in clinical practice. Eighth, although we found that OBS <22 could be used as a risk stratification threshold for high-risk CKM patients, data from clinical trials directly intervening in OBS are lacking, and thus specific strategies to improve OBS and their long-term effects are needed. Finally, the ML model was developed and validated using only internal datasets without external validation, which may limit the generalisability of the findings. Despite interpreting model predictions using SHAP values, a causal relationship could not be established due to the observational design. Future studies should incorporate external datasets and prospective designs to confirm these findings and improve the robustness and clinical applicability of predictive models.

## 5 Conclusion

This study reveals for the first time the negative correlation between OBS and the CKM staging and the mortality risk of CKM patients, with a clear dose-response relationship, emphasizing the clinical value of OBS as a risk stratification and disease management tool. Particularly, OBS below 22 can effectively identify high-risk groups, suggesting the important role of oxidative stress in the poor prognosis of CKM population. Moreover, the LightGBM model demonstrated optimal discriminatory power and predictive accuracy in predicting the risk of advanced CKM staging (AUC for CKM staging: 0.905) and all-cause mortality (AUC for all-cause mortality of CKM: 0.875), with cotinine being the main risk factor. By optimising dietary structure and improving lifestyle, it is promising to enhance the level of OBS, delay the staging of CKM and improve the prognosis of patients, and promote the clinical application of precision chronic disease management. Additionally, future prospective studies should further validate these findings in larger and more diverse cohorts to explore mechanistic pathways underlying the pathophysiology between oxidative stress and CKM.

## CRediT authorship contribution statement

**Yang Chen:** Writing – review & editing, Writing – original draft, Visualization, Validation, Formal analysis, Data curation, Conceptualization. **Shuang Wu:** Writing – review & editing, Visualization, Validation, Formal analysis, Data curation, Conceptualization. **Hongyu Liu:** Writing – review & editing, Visualization, Validation. **Ziyi Zhong:** Writing – review & editing. **Tommaso Bucci:** Writing – review & editing. **Yimeng Wang:** Writing – review & editing. **Manlin Zhao:** Writing – review & editing. **Yang Liu:** Writing – review & editing. **Zhengkun Yang:** Writing – review & editing, Visualization, Validation. **Ying Gue:** Writing – review & editing, Supervision. **Garry McDowell:** Writing – review & editing, Supervision. **Bi Huang:** Writing – review & editing, Supervision, Methodology, Conceptualization. **Gregory Y.H. Lip:** Writing – review & editing, Supervision, Methodology, Conceptualization.

## Funding

No funding towards this work.

## Declaration of competing interest

The authors declare that there are no conflicts of interest associated with this manuscript. All authors have no financial, personal, or professional relationships that could inappropriately influence or bias the content of this work.

## Data Availability

Data will be made available on request.
