# Supplementary material for: Role of oxidative balance score in staging and mortality risk of cardiovascular-kidney-metabolic syndrome: Insights from traditional and machine learning approaches
Source: Redox Biol. 2025 Mar 7;81:103588. doi: 10.1016/j.redox.2025.103588 (PMC11950999; doi:10.1016/j.redox.2025.103588)
Supplement: Multimedia component 1 [file mmc1.docx]

Supplementary Methods. The codes of R and Python used in this study

Supplementary Table S1. Strengthening the Reporting of Observational Studies in Epidemiology Checklist for this cohort study

Supplementary Table S2. Definitions of CKM

Supplementary Table S3. Detailed algorithm of the simplified 10-year CVD risk models

Supplementary Table S4. Detailed algorithm for evaluating each CKM stage

Supplementary Table S5. Oxidative balance score assignment scheme

Supplementary Table S6. Definition of Life’s Simple 7 metrics

Supplementary Table S7. The components and scoring criteria of HEI

Supplementary Table S8. List of 49 variables included in the frailty score

Supplementary Table S9. Proportions of missing value

Supplementary Table S10. Knots selection of restricted cubic spline analysis

Supplementary Table S11. Baseline characteristics of all participants categorized by CKM staging

Supplementary Table S12. Subgroup analysis of exploring the interaction between oxidative balance score and mortality outcomes

Supplementary Table S13. Sensitivity analysis of OBS and mortality outcomes in CKM patients after excluding patients died within the first two-year follow-up

Supplementary Table S14. Sensitivity analysis of OBS and mortality outcomes in CKM patients after excluding patients with cancer

Supplementary Table S15. Association between OBS and mortality outcomes in CKM patients stratified by optimal risk cut-off points

Supplementary Table S16. Best hyperparameters of each machine learning model for predicting advanced staging of cardiovascular-kidney-metabolic syndrome in whole population

Supplementary Table S17. Best hyperparameters of each machine learning model for predicting all-cause mortality in patients with cardiovascular-kidney-metabolic syndrome

Supplementary Table S18. Performance metrics of each machine learning model for predicting advanced CKM staging in whole population

Supplementary Table S19. Performance metrics of each machine learning model for predicting all-cause mortality in CKM patients

Supplementary Figure S1. Flowchart of this study.

Supplementary Figure S2. Variance inflation factor values for features in the model in predicting advanced staging of cardiovascular-kidney-metabolic syndrome.

Supplementary Figure S3. Variance inflation factor values for features in the model in predicting all-cause mortality of patients with cardiovascular-kidney-metabolic syndrome.

Supplementary Figure S4. Distribution of mortality outcomes by CKM stages and OBS quartiles in CKM patients.

Supplementary Figure S5. Kaplan-Meier survival curves for mortality outcomes across OBS quartiles in CKM patients.

Supplementary Figure S6. Restricted cubic spline analyses for associations between OBS and mortality outcomes in CKM patients.

Supplementary Figure S7. Restricted cubic spline analysis of the association between OBS and cardiovascular mortality in patients with cardiovascular-kidney-metabolic syndrom stratified by age (<65 years vs. ≥65 years).

Supplementary Figure S8. Determination of optimal risk stratification cut-off points for OBS on mortality outcomes in CKM patients.

Supplementary Methods. The codes of R and Python used in this study

*Missing value interpolation (Python)*

import miceforest as mf

import pandas as pd

import numpy as np

import seaborn as sns

data = pd.read_csv(' your_file_need_interpolation.csv')

data.isnull().sum()

data_subset = data.iloc[:,:-1]

#last colum in the dataset is the ID, which is used for subsequent data merging

data_subset.isnull().sum()

A_data_missing = mf.ampute_data(data_subset, perc=0.25, random_state=42)

print(A_data_missing.isnull().sum()/len(data_subset))

imputer = mf.ImputationKernel(data=data_subset,

save_all_iterations=True,

random_state=42)

imputer.mice(10, verbose=True)

dataresult=[]

result=[]

for i in range(imputer.dataset_count()):

dataresult.append(imputer.complete_data(i))

dd=((dataresult[i].mean()-A_data_missing.mean()))/A_data_missing.mean()*100

result.append(dd)

print(result)

name=A_data_missing.columns

new_complete=pd.DataFrame(columns=name)

lst=[]

for i in range(len(name)):

re = []

for j in range(imputer.dataset_count()):

re.append(result[j][i])

a=re.index(min(re))

lst.append(a)

for i in range(len(name)):

new_complete[name[i]]=dataresult[lst[i]][name[i]]

print(new_complete)

id_and_outcomes = data.iloc[:, [-1,]]

final_dataset = pd.concat([id_and_outcomes.reset_index(drop=True), new_complete.reset_index(drop=True)], axis=1)

final_dataset.to_csv('/Users/chenyang/Desktop/share_postmice.csv')

*Machine learning modelling (Python)*

import pandas as pd

import numpy as np

import matplotlib.pyplot as plt

import seaborn as sns

import warnings

import shap

import random

import statsmodels.api as sm

import matplotlib as mpl

from statsmodels.stats.outliers_influence import variance_inflation_factor

import matplotlib.pyplot as plt

from collections import Counter

from scipy import stats

from scipy.stats import randint as sp_randint, uniform as sp_uniform

from sklearn.model_selection import train_test_split, GridSearchCV, RandomizedSearchCV

from sklearn.utils import resample

from sklearn.preprocessing import StandardScaler, MinMaxScaler, label_binarize

from sklearn.datasets import make_classification

from sklearn.linear_model import LogisticRegression, RidgeCV, LassoCV, Ridge, Lasso

from sklearn.feature_selection import RFE

from sklearn.preprocessing import StandardScaler

from sklearn.ensemble import RandomForestClassifier

from sklearn.calibration import calibration_curve

from sklearn.multiclass import OneVsRestClassifier

import shap

from sklearn.metrics import (

roc_auc_score, auc, roc_curve, precision_score, recall_score,

f1_score, accuracy_score, classification_report, confusion_matrix,

precision_recall_curve, average_precision_score

)

from hyperopt import fmin, hp, Trials, space_eval, rand, tpe, anneal

import lightgbm as lgb

from imblearn.over_sampling import SMOTE

warnings.filterwarnings("ignore")

df = pd.read_csv('your_files_after_interpolation.csv')

print(df.shape)

df.head(2)

X = df.iloc[:, :-1]

y = df.iloc[:,-1]

X_train, X_test, y_train, y_test = train_test_split(X, y, test_size=0.3,stratify=y,random_state=43)

# For VIF plot

X_train['intercept'] = 1

vif_data = pd.DataFrame()

vif_data['Feature'] = X_train.columns

vif_data['VIF'] = [variance_inflation_factor(X_train.values, i) for i in range(X_train.shape[1])]

X_train = X_train.drop('intercept', axis=1)

vif_data = vif_data[vif_data['Feature'] != 'intercept']

print(vif_data)

plt.figure(figsize=(12, 5))

sns.barplot(x='VIF', y='Feature', data=vif_data, palette='viridis')

plt.title('Variance Inflation Factor (VIF) of Features')

plt.xlabel('VIF')

plt.ylabel('Feature')

plt.savefig('VIF.pdf')

plt.show()

# For LightGBM model

from sklearn.metrics import fbeta_score, make_scorer

param_dist = {

'class_weight':['balanced'],

'num_leaves': randint(20, 200),

'max_depth': randint(5, 12),

'learning_rate': '0.1',

'n_estimators': randint(50, 500),

'min_child_samples': randint(2, 200),

'subsample': uniform(0.5, 0.9),

'colsample_bytree': uniform(0.5, 0.9)

}

lgb_clf = lgb.LGBMClassifier(random_state=42)

scorer = make_scorer(roc_auc_score, greater_is_better=True, needs_proba=True)

random_search = RandomizedSearchCV(estimator=lgb_clf, param_distributions=param_dist, scoring=scorer, n_iter=100, cv=5, random_state=42, n_jobs=-1, verbose=2)

random_search.fit(X_train, y_train)

best_params = random_search.best_params_

best_lgb_clf = lgb.LGBMClassifier(**best_params, random_state=42)

best_lgb_clf.fit(X_train, y_train)

y_train_pred_prob = best_lgb_clf.predict_proba(X_train)[:, 1]

y_test_pred_prob = best_lgb_clf.predict_proba(X_test)[:, 1]

train_auc = roc_auc_score(y_train, y_train_pred_prob)

test_auc = roc_auc_score(y_test, y_test_pred_prob)

model_lgb = lgb.LGBMClassifier(boosting_type='X',

objective='X',

colsample_bytree=X,

learning_rate=X,

max_depth=X,

min_child_samples=X,

n_estimators=X,

num_leaves=X,

subsample=X,

class_weight='balanced')

model_lgb.fit(X_train,y_train)

y_pred_prob_lgb = model_lgb.predict_proba(X_test)[:, 1]

auc_lgb = roc_auc_score(y_test, y_pred_prob_lgb)

print(auc_lgb)

y_pred_lgb = model_lgb.predict(X_test)

f1_lgb = f1_score(y_test, y_pred_lgb)

recall_lgb = recall_score(y_test, y_pred_lgb)

precision_lgb = precision_score(y_test, y_pred_lgb)

accuracy_lgb = accuracy_score(y_test, y_pred_lgb)

n = y_test.shape[0]

z = stats.norm.ppf(0.975) # 95% CI

interval_lgb = z * np.sqrt(auc_lgb * (1 - auc_lgb) / n)

tn_lgb, fp_lgb, fn_lgb, tp_lgb = confusion_matrix(y_test, y_pred_lgb).ravel()

sensitivity_lgb = tp_lgb / (tp_lgb + fn_lgb)

specificity_lgb = tn_lgb / (tn_lgb + fp_lgb)

# For Random Forest model

param_dist = {

'n_estimators': randint(100, 300),

'max_depth': randint(1, 12),

'criterion': ['gini', 'entropy'],

'min_samples_leaf': randint(1, 50),

'min_samples_split': randint(2, 50)

}

rf = RandomForestClassifier(class_weight='balanced',random_state=1)

scorer = make_scorer(roc_auc_score, greater_is_better=True, needs_proba=True)

random_search = RandomizedSearchCV(estimator=rf, param_distributions=param_dist, scoring=scorer, n_iter=100, cv=5, random_state=42, n_jobs=-1, verbose=2)

random_search.fit(X_train, y_train)

best_params = random_search.best_params_

best_rf = RandomForestClassifier(**best_params, random_state=42)

best_rf.fit(X_train, y_train)

y_pred_prob = best_rf.predict_proba(X_test)[:, 1]

best_auc = roc_auc_score(y_test, y_pred_prob)

print(f"Best parameters: {best_params}")

print(f"Best AUC: {best_auc}")

model = RandomForestClassifier(n_estimators=X,random_state=X, class_weight='balanced',

max_depth=X,min_samples_leaf=X,min_samples_split=X,criterion='entropy')

model.fit(X_train,y_train)

y_pred_prob = model.predict_proba(X_test)[:, 1]

auc = roc_auc_score(y_test, y_pred_prob)

print(auc)

n_1 = y_train.shape[0]

z = stats.norm.ppf(0.975)

interval_rf_t = z * np.sqrt(auc_t * (1 - auc_t) / n_1)

y_pred_rf = model.predict(X_test)

f1_rf = f1_score(y_test, y_pred_rf)

recall_rf = recall_score(y_test, y_pred_rf)

precision_rf = precision_score(y_test, y_pred_rf)

accuracy_rf = accuracy_score(y_test, y_pred_rf)

n = y_test.shape[0]

z = stats.norm.ppf(0.975) # 95% CI

interval_rf = z * np.sqrt(auc * (1 - auc) / n)

tn_rf, fp_rf, fn_rf, tp_rf = confusion_matrix(y_test, y_pred_rf).ravel()

sensitivity_rf = tp_rf / (tp_rf + fn_rf)

specificity_rf = tn_rf / (tn_rf + fp_rf)

print(f"AUC: {auc}")

print(f"95% CI: [{auc - interval_rf:.3f}, {auc + interval_rf:.3f}]")

print(f"F1 Score: {f1_rf}")

print(f"Recall: {recall_rf}")

print(f"Precision: {precision_rf}")

print(f"Accuracy: {accuracy_rf}")

print(f"Sensitivity: {sensitivity_rf}")

print(f"Specificity: {specificity_rf}")

# For Logistic Regression model

lr_model = LogisticRegression(solver='liblinear')

param_grid = {

'penalty': ['l1', 'l2'],

'C': [0.01, 0.1, 1, 10, 100],

'solver': ['liblinear', 'saga']

}

grid_search = GridSearchCV(

estimator=lr_model,

param_grid=param_grid,

cv=5,

scoring='accuracy',

n_jobs=-1,

verbose=2

)

grid_search.fit(X_train, y_train)

print("best_params:", grid_search.best_params_)

print("best_score:", grid_search.best_score_)

y_pred = grid_search.predict(X_test)

print("acc in test", accuracy_score(y_test, y_pred))

print("classification report\n", classification_report(y_test, y_pred))

model_lr = LogisticRegression(C=X,penalty='X',solver='X', max_iter=X,class_weight='balanced')

model_lr.fit(X_train,y_train)

y_pred_prob_lr = model_lr.predict_proba(X_test)[:, 1]

auc_lr = roc_auc_score(y_test, y_pred_prob_lr)

print(auc_lr)

y_pred_lr = model_lr.predict(X_test)

f1_lr = f1_score(y_test, y_pred_lr)

recall_lr = recall_score(y_test, y_pred_lr)

precision_lr = precision_score(y_test, y_pred_lr)

accuracy_lr = accuracy_score(y_test, y_pred_lr)

n = y_test.shape[0]

z = stats.norm.ppf(0.975) # 95% CI

interval_lr = z * np.sqrt(auc_lr * (1 - auc_lr) / n)

tn_lr, fp_lr, fn_lr, tp_lr = confusion_matrix(y_test, y_pred_lr).ravel()

sensitivity_lr = tp_lr / (tp_lr + fn_lr)

specificity_lr = tn_lr / (tn_lr + fp_lr)

print(f"AUC: {auc_lr}")

print(f"95% CI: [{auc_lr - interval_lr:.3f}, {auc_lr + interval_lr:.3f}]")

print(f"F1 Score: {f1_lr}")

print(f"Recall: {recall_lr}")

print(f"Precision: {precision_lr}")

print(f"Accuracy: {accuracy_lr}")

print(f"Sensitivity: {sensitivity_lr}")

print(f"Specificity: {specificity_lr}")

# For Support Vector Machine model

scaler = StandardScaler()

features_to_scale = [X'…]

X_train[features_to_scale] = scaler.fit_transform(X_train[features_to_scale])

X_test[features_to_scale] = scaler.transform(X_test[features_to_scale])

smote = SMOTE(random_state=42)

X_train_smote, y_train_smote = smote.fit_resample(X_train, y_train)

svc = SVC()

param_grid = {

'C': [0.1, 1, 10, 100],

'kernel': ['linear', 'rbf', 'poly'],

'gamma': ['scale', 'auto', 0.001, 0.01, 0.1],

'degree': [2, 3, 4]

}

grid_search = GridSearchCV(

estimator=svc,

param_grid=param_grid,

cv=5,

scoring='accuracy',

n_jobs=-1,

verbose=2

)

grid_search.fit(X_train_smote, y_train_smote)

print("best_params:", grid_search.best_params_)

print("best_score", grid_search.best_score_)

y_pred = grid_search.predict(X_test)

print("acc in test", accuracy_score(y_test, y_pred))

print("classification report\n", classification_report(y_test, y_pred))

model_svm = SVC(C=X, kernel='X',gamma='auto',tol=X,probability=True)

model_svm.fit(X_train, y_train)

y_score_svm = model_svm.predict_proba(X_test)[:,1]

auc_svm=roc_auc_score(y_test, y_score_svm)

print(f"AUC: {auc_svm}")

y_pred_svm = model_svm.predict(X_test)

auc_svm = roc_auc_score(y_test, y_score_svm)

f1_svm = f1_score(y_test, y_pred_svm)

recall_svm = recall_score(y_test, y_pred_svm)

precision_svm = precision_score(y_test, y_pred_svm)

accuracy_svm = accuracy_score(y_test, y_pred_svm)

n = y_test.shape[0]

z = stats.norm.ppf(0.975) # 95% CI

interval_svm = z * np.sqrt(auc_svm * (1 - auc_svm) / n)

tn_svm, fp_svm, fn_svm, tp_svm = confusion_matrix(y_test, y_pred_svm).ravel()

sensitivity_svm = tp_svm / (tp_svm + fn_svm)

specificity_svm = tn_svm / (tn_svm + fp_svm)

print(f"AUC: {auc_svm}")

print(f"95% CI: [{auc_svm - interval_svm:.3f}, {auc_svm + interval_svm:.3f}]")

print(f"F1 Score: {f1_svm}")

print(f"Recall: {recall_svm}")

print(f"Precision: {precision_svm}")

print(f"Accuracy: {accuracy_svm}")

print(f"Sensitivity: {sensitivity_svm}")

print(f"Specificity: {specificity_svm}")

# For Multi-layer Perceptron model

scaler = StandardScaler()

features_to_scale = [X'…]

X_train[features_to_scale] = scaler.fit_transform(X_train[features_to_scale])

X_test[features_to_scale] = scaler.transform(X_test[features_to_scale])

smote = SMOTE(random_state=42)

X_train_smote, y_train_smote = smote.fit_resample(X_train, y_train)

param_dist = {

'hidden_layer_sizes': [(10,),(5,),(5,10,),(10,20,),(50,), (100,), (50, 50), (100, 50), (50, 100)],

'activation': ['tanh', 'relu'],

'solver': ['sgd', 'adam'],

'alpha': uniform(0.0001, 0.05),

'learning_rate': ['constant', 'adaptive']

}

mlp_clf = MLPClassifier(max_iter=200, random_state=42)

scorer = make_scorer(roc_auc_score, greater_is_better=True, needs_proba=True)

random_search = RandomizedSearchCV(estimator=mlp_clf, param_distributions=param_dist, scoring=scorer, n_iter=100, cv=5, random_state=42, n_jobs=-1, verbose=2)

random_search.fit(X_train_smote, y_train_smote)

best_params = random_search.best_params_

best_mlp_clf = MLPClassifier(**best_params, max_iter=200, random_state=42)

best_mlp_clf.fit(X_train, y_train)

y_test_pred_prob = best_mlp_clf.predict_proba(X_test)[:, 1]

test_auc = roc_auc_score(y_test, y_test_pred_prob)

print(f"Best parameters: {best_params}")

print(f"Test AUC: {test_auc}")

model_mlp=MLPClassifier(solver='X',activation='X',learning_rate='X',

hidden_layer_sizes=(X,X),alpha=X,max_iter=X,random_state=X)

model_mlp.fit(X_train_smote,y_train_smote)

y_score_mlp = model_mlp.predict_proba(X_test)[:,1]

auc_mlp=roc_auc_score(y_test, y_score_mlp)

print(f"AUC: {auc_mlp}")

y_pred_mlp = model_mlp.predict(X_test)

auc_mlp = roc_auc_score(y_test, y_score_mlp)

f1_mlp = f1_score(y_test, y_pred_mlp)

recall_mlp = recall_score(y_test, y_pred_mlp)

precision_mlp = precision_score(y_test, y_pred_mlp)

accuracy_mlp = accuracy_score(y_test, y_pred_mlp)

n = y_test.shape[0]

z = stats.norm.ppf(0.975) # 95% CI

interval_mlp = z * np.sqrt(auc_mlp * (1 - auc_mlp) / n)

tn_mlp, fp_mlp, fn_mlp, tp_mlp = confusion_matrix(y_test, y_pred_mlp).ravel()

sensitivity_mlp = tp_mlp / (tp_mlp + fn_mlp)

specificity_mlp = tn_mlp / (tn_mlp + fp_mlp)

print(f"AUC: {auc_mlp}")

print(f"95% CI: [{auc_mlp - interval_mlp:.3f}, {auc_mlp + interval_mlp:.3f}]")

print(f"F1 Score: {f1_mlp}")

print(f"Recall: {recall_mlp}")

print(f"Precision: {precision_mlp}")

print(f"Accuracy: {accuracy_mlp}")

print(f"Sensitivity: {sensitivity_mlp}")

print(f"Specificity: {specificity_mlp}")

# Plot ROC

fpr_lgb, tpr_lgb, thresholds_lgb = roc_curve(y_test, y_pred_prob_lgb)

fpr_rf, tpr_rf, thresholds_rf = roc_curve(y_test, y_pred_prob)

fpr_lr, tpr_lr, thresholds_lr = roc_curve(y_test, y_pred_prob_lr)

fpr_svm, tpr_svm, thresholds_svm = roc_curve(y_test, y_score_svm)

fpr_mlp, tpr_mlp, thresholds_mlp = roc_curve(y_test, y_score_mlp)

plt.rcParams.update({'font.family': 'Times New Roman', 'font.size': 12, 'font.weight': 'bold'})

plt.figure(figsize=(12, 9))

plt.plot(fpr_lgb, tpr_lgb, color='darkorange', lw=2,label=f'LightGBM (AUC: X, 95% CI: X-X)')

plt.plot(fpr_rf, tpr_rf, color='olive', lw=2,label=f'RF (AUC: X, 95% CI: X-X)')

plt.plot(fpr_lr, tpr_lr, color='brown', lw=2,label=f'LR (AUC: X, 95% CI: X-X)')

plt.plot(fpr_svm, tpr_svm, color='lightseagreen', lw=2,label=f'SVM (AUC: X, 95% CI: X-X)')

plt.plot(fpr_mlp, tpr_mlp, color='hotpink',lw=2, label=f'MLP (AUC: X, 95% CI: X-X’)

plt.plot([0, 1], [0, 1], color='black', lw=2,linestyle='--')

plt.xlim([0.0, 1.0])

plt.ylim([0.0, 1.05])

plt.xlabel('False Positive Rate',fontsize=18, fontweight='bold')

plt.ylabel('True Positive Rate',fontsize=18, fontweight='bold')

plt.legend(loc="lower right")

plt.title('Receiver Operating Characteristic Curve',fontsize=18, fontweight='bold')

plt.savefig('ROC.pdf')

plt.show()

plt.close()

# Shap plot

explainer = shap.TreeExplainer(XX)

shap_values= explainer.shap_values(X_test)[1]

shap_df = pd.DataFrame(shap_values, columns=X_test.columns)

selected_features = [‘X’,’X’,……]

shap_selected = shap_df[selected_features]

X_selected = X_test[selected_features]

shap.summary_plot(shap_selected.values, X_selected,show=False)

plt.savefig('Shap.pdf')

plt.close()

*Kaplan-Meier survival analysis (R)*

library(rms)

library(gtsummary)

library(survival)

library(ggplot2)

library(ggsci)

data <- read.csv('your_files_after_interpolation.csv')

fit1<-survfit(Surv(follow_up_time, outcome) ~ obs_group, data=data)

ggsurvplot(fit1, data = data,

conf.int=F,

censor=T,

risk.table = TRUE,

pval = sprintf("p = %.4f", surv_pvalue(fit1, data = data)$pval),

xlab = "Time, months",

xlim = c(X,X), ylim = c(X,X),

break.x.by = X)

*Restricted cubic spline analysis (R)*

library(rms)

library(gtsummary)

library(survival)

library(ggplot2)

library(ggsci)

data <- read.csv('your_files_after_interpolation.csv')

refvalue <- X

dd<-datadist(data)

options(datadist='dd')

dd$limits$obs_total[2] <-refvalue

data$X<-factor(data$X)

knots_list <- 3:6

aic_bic_results <- data.frame(Knots = integer(), AIC = numeric(), BIC = numeric())

for (k in knots_list) {

cox_model <- coxph(Surv(follow_up_time, outcome) ~ ns(obs, df = k) + covariate, data = data)

aic_value <- AIC(cox_model)

bic_value <- BIC(cox_model)

aic_bic_results <- rbind(aic_bic_results, data.frame(Knots = k, AIC = aic_value, BIC = bic_value))

}

print(aic_bic_results)

#Based on the results of BIC (priority) and AIC, select the optimal knots number

Fit_rcs <- cph(Surv(follow_up_time, outcome) ~ rcs(obs,knots)+ covariate,

x=TRUE, y=TRUE,data=data)

Pre_rcs <-rms::Predict(fit_rcs,obs,fun=exp,type="predictions",ref.zero=TRUE,conf.int = 0.95,digits=2);

ggplot() +

geom_line(data = Pre_rcs,

aes(obs, yhat),

linetype = 1,

alpha = 0.7,

colour = 'red',

linewidth = 1) +

scale_color_nejm() +

geom_ribbon(data = Pre_rcs,

aes(obs, ymin = lower, ymax = upper),

alpha = 0.1, fill = 'red') +

scale_fill_nejm() +

theme_classic() +

geom_hline(yintercept = 1, linetype = 2, linewidth = 0.75) +

labs(title = "",

x = "OBS",

y = "HR (95% CI)") +

geom_point(aes(x = refvalue, y = 1), colour = "red", size = 3)

*Mediation analysis (R)*

library(mediation)

data <- read.csv('your_files_after_interpolation.csv')

data$X<-factor(data$X)

set.seed(12345)

a <- glm(outcome ~ independent_variable + covariate, data=data,family = binomial("probit"))

summary(a)

b <- lm(obs ~ independent_variable + covariate data=data)

summary(b)

c <- glm(outcome ~ independent_variable + obs + covariate, data=data,family = binomial("probit"))

summary(c)

contcont <- mediate(b, c, sims=1000, treat=" independent_variable ", mediator="obs ")

summary(contcont)

*Analysis for identifying optimal cut-off point (R)*

library(survminer)

data <- read.csv('your_files_after_interpolation.csv')

data$X<-factor(data$X)

fit_cutoff <- coxph(Surv(follow_up_time, outcome) ~ obs + covirate, data=data)

res.cut<-surv_cutpoint(data, time=" follow_up_time ", event="outcome ", variables = c("obs "))

summary(res.cut)

plot(res.cut,"obs",palette="npg")

Supplementary Table S1. Strengthening the Reporting of Observational Studies in Epidemiology Checklist for this cohort study

|  | Item No | Recommendation | Page No |
| --- | --- | --- | --- |
| **Title and abstract** | 1 | (*a*) Indicate the study’s design with a commonly used term in the title or the abstract | 1,3 |
|  |  | (*b*) Provide in the abstract an informative and balanced summary of what was done and what was found | 3 |
| Introduction | | | |
| Background/rationale | 2 | Explain the scientific background and rationale for the investigation being reported | 5,6 |
| Objectives | 3 | State specific objectives, including any prespecified hypotheses | 6 |
| Methods | | | |
| Study design | 4 | Present key elements of study design early in the paper | 7,8,9 |
| Setting | 5 | Describe the setting, locations, and relevant dates, including periods of recruitment, exposure, follow-up, and data collection | 6,7,8,9 |
| Participants | 6 | (*a*) Give the eligibility criteria, and the sources and methods of selection of participants. Describe methods of follow-up | 7,8 |
|  |  | (*b*) For matched studies, give matching criteria and number of exposed and unexposed | N/A |
| Variables | 7 | Clearly define all outcomes, exposures, predictors, potential confounders, and effect modifiers. Give diagnostic criteria, if applicable | 7,8,9 |
| Data sources/ measurement | 8* | For each variable of interest, give sources of data and details of methods of assessment (measurement). Describe comparability of assessment methods if there is more than one group | 6,7 |
| Bias | 9 | Describe any efforts to address potential sources of bias | 10,11,12 |
| Study size | 10 | Explain how the study size was arrived at | N/A |
| Quantitative variables | 11 | Explain how quantitative variables were handled in the analyses. If applicable, describe which groupings were chosen and why | 8,9 |
| Statistical methods | 12 | (*a*) Describe all statistical methods, including those used to control for confounding | 9,10,11,12 |
|  |  | (*b*) Describe any methods used to examine subgroups and interactions | 10 |
|  |  | (*c*) Explain how missing data were addressed | 9 |
|  |  | (*d*) If applicable, explain how loss to follow-up was addressed | 7 |
|  |  | (*e*) Describe any sensitivity analyses | 11 |
| Results | | |  |
| Participants | 13* | (a) Report numbers of individuals at each stage of study—eg numbers potentially eligible, examined for eligibility, confirmed eligible, included in the study, completing follow-up, and analysed | 13 |
|  |  | (b) Give reasons for non-participation at each stage | 7 |
|  |  | (c) Consider use of a flow diagram | 7 |
| Descriptive data | 14* | (a) Give characteristics of study participants (eg demographic, clinical, social) and information on exposures and potential confounders | 12,13 |
|  |  | (b) Indicate number of participants with missing data for each variable of interest | 7,9 |
|  |  | (c) Summarise follow-up time (eg, average and total amount) | 12 |
| Outcome data | 15* | Report numbers of outcome events or summary measures over time | 13,14 |
| Main results | 16 | (*a*) Give unadjusted estimates and, if applicable, confounder-adjusted estimates and their precision (eg, 95% confidence interval). Make clear which confounders were adjusted for and why they were included | 14,15 |
|  |  | (*b*) Report category boundaries when continuous variables were categorized | 14,15 |
|  |  | (*c*) If relevant, consider translating estimates of relative risk into absolute risk for a meaningful time period | 14,15 |
| Other analyses | 17 | Report other analyses done—eg analyses of subgroups and interactions, and sensitivity analyses | 15,16,17 |
| Discussion | | | |
| Key results | 18 | Summarise key results with reference to study objectives | 17 |
| Limitations | 19 | Discuss limitations of the study, taking into account sources of potential bias or imprecision. Discuss both direction and magnitude of any potential bias | 22,23 |
| Interpretation | 20 | Give a cautious overall interpretation of results considering objectives, limitations, multiplicity of analyses, results from similar studies, and other relevant evidence | 18,19,20,21,22 |
| Generalisability | 21 | Discuss the generalisability (external validity) of the study results | 20,21,22 |
| Other information | | | |
| Funding | 22 | Give the source of funding and the role of the funders for the present study and, if applicable, for the original study on which the present article is based | 25 |

*Give information separately for exposed and unexposed groups

**Note:** An Explanation and Elaboration article discusses each checklist item and gives methodological background and published examples of transparent reporting. The STROBE checklist is best used in conjunction with this article (freely available on the Web sites of PLoS Medicine at http://www.plosmedicine.org/, Annals of Internal Medicine at http://www.annals.org/, and Epidemiology at http://www.epidem.com/). Information on the STROBE Initiative is available at http://www.strobe-statement.org

Supplementary Table S2. Definitions of CKM

| CKM conditions | Definition | CKM indicators | Threshold for CKM indicators |
| --- | --- | --- | --- |
| CVD | Individuals with clinical CVD or subclinical CVD | Clinical CVD | History of chronic heart failure, coronary heart disease, heart attack, or stroke |
|  |  | Subclinical CVD | Any of the following criterion is met:  1) Very high-risk CKD in KDIGO classification: UACR ≥ 300 mg/g and eGFR ≤ 45-59 ml/min/1.73m2, UACR ≥ 30 mg/g and eGFR ≤ 30-44 ml/min/1.73m2, or eGFR ≤ 29 ml/min/1.73m2.  2) Predicted 10-year CVD risk ≥ 20% |
| Kidney diseases | Individuals with CKD | CKD | Moderate-to-high-risk CKD in KDIGO classification: UACR ≥ 30 mg/g and eGFR ≥ 60 ml/min/1.73m2, UACR < 300 mg/g and eGFR ≤ 45-59 ml/min/1.73m2, or UACR < 30 mg/g and eGFR ≤ 30-44 ml/min/1.73m2. |
| Metabolic disorders | Individuals with overweight/obesity, abdominal obesity, prediabetes, diabetes, hypertension, hypertriglyceridemia or MetS | Overweight/obesity | BMI ≥25 kg/m2 (or ≥23 kg/m2 if Asian ancestry)* |
|  |  | Abdominal obesity | Waist circumference ≥88/102 cm in female/male (or if Asian ancestry ≥80/90 cm in female/male) |
|  |  | Prediabetes | Fasting blood glucose ≥ 100-124 mg/dL or HbA1c ≥ 5.7%-6.4% and without self-reported diagnosis of diabetes, use of insulin, or oral hypoglycemic agents |
|  |  | Diabetes | Fasting blood glucose ≥ 125 mg/dL or HbA1c ≥ 6.5% or self-reported diagnosis of diabetes, use of insulin, or oral hypoglycemic agents |
|  |  | Hypertension | SBP ≥130 mm Hg or DBP ≥80 mm Hg or self-reported diagnosis of hypertension or use of antihypertensive medications |
|  |  | Hypertriglyceridemia | Triglycerides ≥ 135 mg/dL |
|  |  | MetS | MetS is defined by the presence of 3 or more of the following:   \| 1) Waist circumference ≥88/102 cm in female/male (or if Asian ancestry ≥80/90 cm in female/male).  2) HDL cholesterol <50/40 mg/dL in female/male.  3) Triglycerides ≥150 mg/dL.  4) Elevated blood pressure (SBP ≥130 mm Hg or DBP ≥80 mm Hg and/or use of antihypertensive medications).  5) Fasting blood glucose ≥100 mg/dL. \| \| --- \| |

*Asian was not listed as a separate race/ethnicity until NAHNES 2011-2012, therefore the uniform threshold for BMI and waist circumference was used in all participants in NHANES 1999-2010.

Abbreviations: BMI, body mass index; CKD, chronic kidney disease; CKM, cardiovascular-kidney-metabolic syndrom; CVD, cardiovascular disease; DBP, diastolic blood pressure; eGFR, estimated glomerular filtration rate; HDL-C, high-density lipoprotein cholesterol; KDIGO, The Kidney Disease: Improving Global Outcomes; MetS, metabolic syndrome; SBP, systolic blood pressure; UACR, urinary albumin to creatinine ratio.

Supplementary Table S3. Detailed algorithm of the simplified 10-year cardiovascular disease risk models

| Sex | Calculation |
| --- | --- |
| Women | log-Odds = -3.307728 + 0.7939329 × (age – 55) /10 + 0.0305239 × ((TC – HDL-C) × 0.02586 – 3.5) – 0.1606857 × (HDL-C – 1.3) /0.3 – 0.2394003 × (min(SBP, 110) – 110) /20 + 0.360078 × (max(SBP, 110) – 130) /20 + 0.8667604 × (if diabetes) + 0.5360739 × (if current smoker) + 0.6045917 × (min(eGFR, 60) – 60) / -15 + 0.0433769 × (max(eGFR, 60) – 90) / -15 + 0.3151672 × (if using anti-hypertensive medication) – 0.1477655 × (if using statin) – 0.0663612 × (if using anti-hypertensive medication) × (max(SBP, 110) – 130) /20 + 0.1197879 × (if using statin) × (TC – HDL-C – 3.5) – 0.0819715 × (age – 55) /10 × (TC – HDL-C – 3.5) + 0.0306769 × (age – 55) /10 × (HDL-C – 1.3) /0.3 – 0.0946348 × (age – 55) /10 × (max(SBP, 110) – 130) /20 – 0.27057 × (age – 55) /10 × (if diabetes) – 0.078715 × (age – 55) /10 × (if current smoker) – 0.1637806 × (age – 55) /10 × (min(eGFR, 60) – 60) / -15  Risk = exp(log-Odds) / (1 + exp(log-Odds)) |
| Men | log-Odds = -3.031168 + 0.7688528 × (age – 55) /10 + 0.0736174 × ((TC – HDL-C) × 0.02586 – 3.5) – 0.0954431 × (HDL-C – 1.3) /0.3 – 0.4347345 × (min(SBP, 110) – 110) /20 + 0.3362658 × (max(SBP, 110) – 130) /20 + 0.7692857 × (if diabetes) + 0.4386871 × (if current smoker) + 0.5378979 × (min(eGFR, 60) – 60) / -15 + 0.0164827 × (max(eGFR, 60) – 90) / -15 + 0.288879 × (if using anti-hypertensive medication) – 0.1337349 × (if using statin) – 0.0475924 × (if using anti-hypertensive medication) × (max(SBP, 110) – 130) /20 + 0.150273 × (if using statin) × (TC – HDL-C – 3.5) – 0.0517874 × (age – 55) /10 × (TC – HDL-C – 3.5) + 0.0191169 × (age – 55) /10 × (HDL-C – 1.3) /0.3 – 0.1049477 × (age – 55) /10 × (max(SBP, 110) – 130) /20 – 0.2251948 × (age – 55) /10 × (if diabetes) – 0.0895067 × (age – 55) /10 × (if current smoker) – 0.1543702 × (age – 55) /10 × (min(eGFR, 60) – 60) / -15  Risk = exp(log-Odds) / (1 + exp(log-Odds)) |

Age extraction: Data File: DEMO.xpt~DEMO_J.xpt; Component: RIDAGEYR

Proportion of missing value: no missing value

Sex extraction: Data File: DEMO.xpt~DEMO_J.xpt; Component: RIAGENDR

Proportion of missing value: no missing value

TC extraction:1999-2000: Data File: LAB13.xpt; Component: LBXTC

2001-2002: Data File: l13_b.xpt, l13_2_b.xpt; Component: LBXTC, LB2TC

2003-2004: Data File: l13_c.xpt; Component: LBXTC

2005-2018: Data File: TCHOL_D.xpt ~ TCHOL_J.xpt; Component: LBXTC

Proportion of missing value: 6 (0.03%)

HDL extraction: 1999-2000: Data File: LAB13.xpt; Component: LBDHDL

2001-2002: Data File: l13_b.xpt, l13_2_b.xpt; Component: LBDHDL, LB2HDL

2003-2004: Data File: l13_c.xpt; Component: LBDHDL

2005-2018: Data File: HDL_D.xpt ~ HDL_J.xpt; Component: LBDHDD

Proportion of missing value: 7 (0.03%)

SBP extraction: Data File: BPX.xpt~BPX_J.xpt; Component: BPXSY1, BPXSY2, BPXSY3, BPXSY4

Proportion of missing value: 64 (0.30%)

Antihypertension medication extraction: Data File: BPQ.xpt~BPQ_J.xpt; Component: BPQ040A

Proportion of missing value: 12 (0.06%)

Statin extraction: Data File: BPQ.xpt~BPQ_J.xpt; Component: BPQ090D

Proportion of missing value: 12 (0.06%)

Smoke extraction: Data File: SMQ.xpt~SMQ_J.xpt; Component: SMD020, SMD040

Proportion of missing value: 13 (0.06%)

Diabetes extraction: Data File: DIQ.xpt~DIQ_J.xpt; Component: DIQ010

Proportion of missing value: no missing value

Extraction of serum creatinine needed to calculate eGFR: 1999-2000: Data File: LAB18.xpt; Component: LBXSCR

2001-2002: Data File: L40_B.xpt, L40_2_B.xpt; Component: LBDSCR, LB2SCR

2003-2004: Data File: L40_C.xpt; Component: LBXSCR

2005-2018: Data File: BIOPRO_D.xpt ~ BIOPRO_J.xpt; Component: LBXSCR

Proportion of missing value: 11 (0.05%)

This assessment was consistent with the procedure described by Khan et al. (Reference: Khan, S. S., Coresh, J., Pencina, M. J., Ndumele, C. E., Rangaswami, J., Chow, S. L., Palaniappan, L. P., Sperling, L. S., Virani, S. S., Ho, J. E., Neeland, I. J., Tuttle, K. R., Rajgopal Singh, R., Elkind, M. S. V., Lloyd-Jones, D. M., & American Heart Association (2023). Novel Prediction Equations for Absolute Risk Assessment of Total Cardiovascular Disease Incorporating Cardiovascular-Kidney-Metabolic Health: A Scientific Statement From the American Heart Association. Circulation, 148(24), 1982–2004. https://doi.org/10.1161/CIR.0000000000001191).

Among the study participants, 77 individuals had missing values for the 10-year cardiovascular risk score. However, these participants could still be clearly classified as stage 3 or 4 of cardiovascular-kidney-metabolic syndrom based on other available variables.

Abbreviations: eGFR, estimated glomerular filtration rate; HDL-C, high-density lipoprotein cholesterol; SBP, systolic blood pressure; TC, total cholesterol.

Supplementary Table S4. Detailed algorithm for evaluating each CKM stage

| CKM stages | Definition | Criterion | Threshold for CKM conditions |
| --- | --- | --- | --- |
| Stage 0: No CKM risk factors | Individuals with normal BMI and waist circumference, normoglycemia, normotension, a normal lipid profile, and no evidence of CKD or subclinical or clinical CVD | All criteria are met | BMI <25 kg/m2 (or <23 kg/m2 if Asian ancestry)* |
|  |  |  | Waist circumference <88/102 cm in female/male (or if Asian ancestry <80/90 cm in female/male) |
|  |  |  | Fasting blood glucose < 100 mg/dL and HbA1c < 5.7% and without self-reported diagnosis of diabetes, use of insulin, or oral hypoglycemic agents |
|  |  |  | SBP <130 mm Hg and DBP <80 mm Hg without self-reported diagnosis of hypertension or use of antihypertensive medications |
|  |  |  | HDL cholesterol >50/40 mg/dL in female/male and triglycerides < 150 mg/dL |
|  |  |  | Low-risk CKD in KDIGO classification according to eGFR and UACR: UACR < 30 mg/g and eGFR ≥ 60 ml/min/1.73m2. |
|  |  |  | Predicted 10-year CVD risk < 20% |
|  |  |  | No clinical CVD |
| Stage 1: Excess or dysfunctional adiposity | Individuals with overweight/obesity, abdominal obesity, or dysfunctional adipose tissue, without the presence of other metabolic risk factors or CKD | Any of the three criteria is met | Overweight/obesity |
|  |  |  | Abdominal obesity |
|  |  |  | Prediabetes |
|  |  | All criteria are met | SBP <130 mm Hg and DBP <80 mm Hg without self-reported diagnosis of hypertension or use of antihypertensive medications |
|  |  |  | HDL cholesterol >50/40 mg/dL in female/male and triglycerides <150 mg/dL |
|  |  |  | Low-risk CKD in KDIGO classification according to eGFR and UACR: UACR < 30 mg/g and eGFR ≥ 60 ml/min/1.73m2 |
|  |  |  | Predicted 10-year CVD risk < 20% |
|  |  |  | No clinical CVD |
| Stage 2: Metabolic risk factors and CKD | Individuals with metabolic risk factors (hypertriglyceridemia, hypertension, MetS, diabetes), or CKD | Any of the five criteria is met | Hypertriglyceridemia |
|  |  |  | Hypertension |
|  |  |  | diabetes |
|  |  |  | MetS |
|  |  |  | Moderate-to-high-risk CKD in KDIGO classification |
|  |  | All criteria are met | No very high-risk CKD in KDIGO classification |
|  |  |  | Predicted 10-year CVD risk < 20% |
|  |  |  | No clinical CVD |
| Stage 3: Subclinical CVD in CKM | Subclinical CVD among individuals with excess/dysfunctional adiposity, other metabolic risk factors, or CKD | Any of the two criteria is met | Very high-risk CKD in KDIGO classification |
|  |  |  | Predicted 10-year CVD risk ≥ 20% |
|  |  | Any of the eight criteria is met | Overweight/obesity |
|  |  |  | Abdominal obesity |
|  |  |  | Prediabetes |
|  |  |  | Hypertriglyceridemia |
|  |  |  | Hypertension |
|  |  |  | diabetes |
|  |  |  | MetS |
|  |  |  | Moderate-to-high-risk CKD in KDIGO classification |
|  |  | The criterion is met | No clinical CVD |
| Stage 4: Clinical CVD in CKM | Clinical CVD among individuals with excess/dysfunctional adiposity, other metabolic risk factors, or CKD | The criterion is met | Clinical CVD |
|  |  | Any of the nine criteria is met | Overweight/obesity |
|  |  |  | Abdominal obesity |
|  |  |  | Prediabetes |
|  |  |  | Hypertriglyceridemia |
|  |  |  | Hypertension |
|  |  |  | diabetes |
|  |  |  | MetS |
|  |  |  | Moderate-to-high-risk CKD in KDIGO classification |
|  |  |  | Very high-risk CKD in KDIGO classification |

*Asian was not listed as a separate race/ethnicity until NHANES 2011-2012, therefore the uniform threshold for BMI and waist circumference was used in all participants in NHANES 1999-2010.

Among the study participants, 77 individuals had missing values for the 10-year CVD risk score. However, these participants could still be clearly classified as stage 3 or 4 of cardiovascular-kidney-metabolic syndrom based on other available variables.

Abbreviations: BMI, body mass index; CKD, chronic kidney disease; CKM, cardiovascular-kidney-metabolic syndrom; CVD, cardiovascular disease; DBP, diastolic blood pressure; eGFR, estimated glomerular filtration rate; HDL-C, high-density lipoprotein; KDIGO, The Kidney Disease: Improving Global Outcomes; NHANES, National Health and Nutrition Examination Survey; SBP, systolic blood pressure; UACR, urinary albumin to creatinine ratio.

Supplementary Table S5. Oxidative balance score assignment scheme

| OBS components | Property | Male | | | Female | | |
| --- | --- | --- | --- | --- | --- | --- | --- |
|  |  | 0 | 1 | 2 | 0 | 1 | 2 |
| Dietary fiber (g/d) | Antioxidant | <14.25 | 14.25–21.20 | ≥21.20 | <12.25 | 12.25–18.00 | ≥18.00 |
| β-Carotene (RE/d) | Antioxidant | <710.00 | 710.00–2178.50 | ≥2178.50 | <839.50 | 839.50–2371.00 | ≥2371.00 |
| Vitamin B2 (mg/d) | Antioxidant | <2.06 | 2.06–3.41 | ≥3.41 | <1.68 | 1.68–3.04 | ≥3.04 |
| Niacin (mg/d) | Antioxidant | <24.82 | 24.82–40.32 | ≥40.32 | <18.64 | 18.64–33.92 | ≥33.92 |
| Vitamin B6 (mg/d) | Antioxidant | <1.67 | 1.67–2.39 | ≥2.39 | <1.31 | 1.31–1.90 | ≥1.90 |
| Total folate (mcg/d) | Antioxidant | <389.50 | 389.50–903.50 | ≥903.50 | <322.00 | 322.00–938.50 | ≥938.5 |
| Vitamin B12 (mcg/d) | Antioxidant | <4.62 | 4.62–15.14 | ≥15.14 | <3.71 | 3.71–15.33 | ≥15.33 |
| Vitamin C (mg/d) | Antioxidant | <39.25 | 39.25–109.935 | ≥109.935 | <40.41 | 40.41–115.32 | ≥115.32 |
| Vitamin E (ATE) (mg/d) | Antioxidant | <6.20 | 6.20–9.49 | ≥9.49 | <5.25 | 5.25–8.24 | ≥8.24 |
| Calcium (mg/d) | Antioxidant | <771.50 | 771.50–1219.00 | ≥1219.00 | <802.50 | 802.50–1362.00 | ≥1362.00 |
| Magnesium (mg/d) | Antioxidant | <274.00 | 274.00–386.00 | ≥386.00 | <234.00 | 234.00–325.50 | ≥325.50 |
| Zinc (mg/d) | Antioxidant | <11.12 | 11.12–19.73 | ≥19.73 | <8.58 | 8.58–17.78 | ≥17.78 |
| Copper (mg/d) | Antioxidant | <1.18 | 1.18–1.85 | ≥1.85 | <1.02 | 1.02–1.62 | ≥1.62 |
| Selenium (mcg/d) | Antioxidant | <108.10 | 108.10–164.20 | ≥164.20 | <81.90 | 81.90–124.90 | ≥124.90 |
| Total fat (g/d) | Prooxidant | ≥90.18 | 61.50–90.18 | <61.50 | ≥71.90 | 47.26–71.90 | <47.26 |
| Iron (mg/d) | Prooxidant | ≥19.70 | 12.66–19.70 | <12.66 | ≥16.99 | 10.65–16.99 | <10.65 |
| Physical activity (MET-min/wk) | Antioxidant | <150 | 150–300 | ≥300 | <150 | 150–300 | ≥300 |
| Alcohol (drinks/d) | Prooxidant | ≥2 drinks/d | < 2 drinks/d | <12 drinks/y | ≥1 drinks/d | < 1 drinks/d | <12 drinks/y |
| Body mass index (kg/m^2^) | Prooxidant | ≥30.10 | 26.00–30.10 | <26.00 | ≥31.40 | 25.80–31.40 | <25.80 |
| Cotinine (ng/mL) | Prooxidant | ≥0.062 | 0.011–0.062 | <0.011 | ≥0.036 | 0.011–0.036 | <0.011 |

Total intake dietary plus supplement intakes; inclusion of supplemental intake based on the availability of supplemental intake information.

Abbreviations: OBS, oxidative balance score; RE, retinol equivalent; ATE, alpha-tocopherol equivalent; MET, metabolic equivalent.

Supplementary Table S6. Definition of Life’s Simple 7 metrics^a^

| Metric | Level of Cardiovascular Health | | | Variables/Files in NHANE | Proportion of missing value, n (%) |
| --- | --- | --- | --- | --- | --- |
|  | Poor health (score = 0) | Intermediate health  (score = 1) | Ideal health  (score =2) |  |  |
| Blood pressure | BP ≥140/90 mmHg | SBP 120 to 139 mmHg or DBP 80 to 89 mmHg or treated to <120/80 mmHg | <120/80 mmHg and not on antihypertensive medication | SBP:  Data File: BPX.xpt~BPX_J.xpt; Component: BPXSY1, BPXSY2, BPXSY3, BPXSY4    DBP:  Data File: BPX.xpt~BPX_J.xpt; Component: BPXDI1, BPXDI2, BPXDI3, BPXDI4    Antihypertension medication:  Data File: BPQ.xpt~BPQ_J.xpt; Component: BPQ040A | 71 (0.33%) |
| Glycemic status ^b^ | HbA1c ≥6.5% | HbA1c 5.7% to 6.4% or treated with insulin or diabetic pills to lower blood sugar to HbA1c <5.7% | HbA1c <5.7% and not on glucose-lowering medication | HbA1c:  1999-2000: Data File: LAB10.xpt; Component: LBXGH;  2001-2004: Data File: L10_B~L10_C.xpt; Component: LBXGH;  2005-2018: Data File: GHB_D~GHB_J.xpt; Component: LBXGH  Treatment with insulin:  Data File: DIQ.xpt~DIQ_J.xpt; Component: DIQ050  Treatment with diabetic pills to lower blood sugar:  1999-2004: Data File: DIQ.xpt ~ DIQ_C.xpt; Component: DIQ070  2005-2008: Data File: DIQ_D.xpt ~ DIQ_E.xpt; Component: DID070  2009-2018: Data File: DIQ_F.xpt ~ DIQ_J.xpt; Component: DIQ070 | 288 (1.33%) |
| Total cholesterol | ≥240 mg/dL | 200 to 239 mg/dL or treated to <200 mg/dL | <200 mg/dL and not on lipid-lowering medication | Total cholesterol:  1999-2000: Data File: LAB13.xpt; Component: LBXTC  2001-2002: Data File: l13_b.xpt, l13_2_b.xpt; Component: LBXTC, LB2TC  2003-2004: Data File: l13_c.xpt; Component: LBXTC  2005-2018: Data File: TCHOL_D.xpt ~ TCHOL_J.xpt; Component: LBXTC  Lipid-lowering medication:  Data File: BPQ.xpt~BPQ_J.xpt; Component: BPQ090D | 6 (0.03%) |
| BMI | ≥30 kg/m^2^ | 25 to 29.9 kg/m^2^ | < 25 kg/m^2^ | Data File: BMX.xpt~BMX_J.xpt; Component: BMXBMI | No missing value |
| Smoking ^c^ | Current smoker who had smoked ≥ 100 cigarettes in their lifetime and currently smoke some days or every day | Former smoker who had smoked ≥100 cigarettes in their lifetime but did not currently smoke | Never smoker or had smoked < 100 cigarettes in their lifetime | SMQ.xpt~SMQ_J.xpt; Component: SMD020, SMD040 | 13 (0.06%) |
| Diet | HEI-2020 <50 | HEI-2020 50 to 80 | HEI-2020 >80 | refer to HEI-2020 score | 3 (0.01%) |
| Physical activity | No activity | 1 to 149 minutes moderate/vigorous per week | ≥150 minutes moderate/vigorous per week | 1999-2006: Data File: PAQ.xpt ~ PAQ_D.xpt; Component: PAQ050Q, PAD080, PAD120, PAD160  2007-2018: Data File: PAQ_H.xpt ~ PAQ_J.xpt; Component: PAQ605, PAQ615, PAQ620, PAQ630, PAQ635, PAQ645, PAQ650, PAQ660, PAQ665, PAD675 | 557 (2.58%) |

^a^The AHA definitions for poor, intermediate, and ideal health were used for blood pressure, cholesterol, BMI, and physical activity; modified definitions were used for glycemic status, smoking, and diet in National Health and Nutrition Examination Survey.

^b^Glycemic status: AHA defined poor health as FPG ≥ 126 mg/dL or HbA1c ≥ 7%, intermediate health as FPG 100 to 125 mg/dL or HbA1c < 7%, and ideal health as FPG < 100 mg/dL.

^c^Smoking: AHA defined poor health as current smoker, intermediate health as quit smoking < 12 months, and ideal health as never smoker or quit smoking ≥ 12 months.

Each component of the LS7 metrics was assigned a score of 2 points for ideal health, 1 point for intermediate health, and 0 point for poor health.

This assessment was consistent with the procedure described by Brown et al. (Reference: Brown, A. F., Liang, L. J., Vassar, S. D., Escarce, J. J., Merkin, S. S., Cheng, E., Richards, A., Seeman, T., & Longstreth, W. T., Jr (2018). Trends in Racial/Ethnic and Nativity Disparities in Cardiovascular Health Among Adults Without Prevalent Cardiovascular Disease in the United States, 1988 to 2014. Annals of internal medicine, 168(8), 541–549. https://doi.org/10.7326/M17-0996)

Abbreviations: BP, blood pressure; SBP, systolic blood pressure; DBP, diastolic blood pressure; HbA1c, hemoglobin A1c; BMI, body mass index; HEI-2020, Healthy Eating Index-2020; AHA, American Heart Association; FPG, fasting plasma glucose.

Supplementary Table S7. The components and scoring criteria of HEI-2020

| HEI Components | Range of Points | Minimum Scoring Standard | Maximum Scoring Standard | Proportion of missing value, n (%) |
| --- | --- | --- | --- | --- |
| Adequacy Components  (higher score indicates higher consumption) | | | |  |
| Total Fruits | 0-5 | 0 | 0.8 cup equiv. /1000 kcal | No missing value |
| Whole Fruits | 0-5 | 0 | 0.4 cup equiv./1000 kcal | No missing value |
| Total Vegetables | 0-5 | 0 | 1.1 cup equiv. /1000 kcal | No missing value |
| Greens and Beans | 0-5 | 0 | 0.2 cup equiv. /1000 kcal | No missing value |
| Total Protein Foods | 0-5 | 0 | 2.5 oz equiv./1000 kcal | No missing value |
| Seafood and Plant Proteins | 0-5 | 0 | 0.8 oz equiv./1000 kcal | No missing value |
| Dairy | 0-10 | 0 | 1.3 cup equiv./1000 kcal | No missing value |
| Whole Grains | 0-10 | 0 | 1.5 oz equiv. /1000 kcal | No missing value |
| Fatty Acids ^b^ | 0-10 | (PUFAs + MUFAs)/SFAs ≤1.2 | (PUFAs + MUFAs)/SFAs ≥2.5 | 3 (0.01%) |
| Moderation Components  (higher score indicates lower consumption) | | | |  |
| Refined Grains | 0-10 | 4.3 oz equiv./1000 kcal | 1.8 oz equiv. /1000 kcal | No missing value |
| Sodium | 0-10 | 2.0 grams /1000 kcal | 1.1 grams/1000 kcal | No missing value |
| Added Sugars | 0-10 | 26% of energy | 6.5% of energy | No missing value |
| Saturated Fats | 0-10 | 16% of energy | 8% of energy | No missing value |

^a^Intakes between the minimum and maximum standards are scored proportionately.

^b^Ratios of polyunsaturated and monounsaturated fatty acids (PUFAs and MUFAs) to saturated fatty acids (SFAs).

HEI-2020 calculations were done based on files of Food Patterns Equivalents Database (FPED) and the R package ‘dietaryindex’. The FPED serves as a unique research tool to evaluate food and beverage intakes of Americans with respect to the 2015-2020 Dietary Guidelines for Americans recommendations (https://www.ars.usda.gov/ northeast-area/beltsville-md-bhnrc/beltsville-human-nutrition-research-center/food-surveys-research-group/docs/fped-databases/). The ‘dietaryindex’ is an R package to standardise the compilation of dietary intake data into index-based dietary patterns to enable the assessment of adherence to these patterns in epidemiologic and clinical studies. It has been peer-reviewed and published in the American Journal of Clinical Nutrition (Zhan, J. J., Hodge, R. A., Dunlop, A. L., Lee, M. M., Bui, L., Liang, D., & Ferranti, E. P. (2024). Dietaryindex: a user-friendly and versatile R package for standardizing dietary pattern analysis in epidemiological and clinical studies. The American journal of clinical nutrition, 120(5), 1165–1174. https://doi.org/10.1016/j.ajcnut.2024.08.021). The specific HEI-2020 calculation process and R code are detailed at https://github.com/jamesjiadazhan/dietaryindex.

The NHANES source files required are: DRXTOT, DRXTOT_B, DR1TOT_C, DR2TOT_C, DR1TOT_D, DR2TOT_D, DR1TOT_E, DR2TOT_E, DR1TOT_F, DR2TOT_F, DR1TOT_G, DR2TOT_G, DR1TOT_H, DR2TOT_H, DR1TOT_I, DR2TOT_I, DR1TOT_J, DR2TOT_J.

Development and validation of HEI-2020 were available at https://epi.grants.cancer.gov/hei/developing.html#2015

Abbreviations: HEI-2020, Healthy Eating Index-2020; PUFAs, polyunsaturated monounsaturated fatty acids; MUFAs, monounsaturated fatty acids; SFAs, saturated fatty acids.

Supplementary Table S8. List of 49 variables included in the frailty score

| Variable | Scoring | Variable/File in NHANES | Proportion of missing value, n (%) |
| --- | --- | --- | --- |
| Cognition |  |  |  |
| 1. Experience confusion/memory problems | Yes = 1;  No = 0 | 1999-2000: Data File: PFQ.xpt;  Component: PFQ056  2001-2002: Data File: PFQ_B.xpt;  Component: PFQ056  2003-2018: Data File: PFQ_C.xpt~PFQ_J.xpt;  Component: PFQ057 | 10 (0.05%) |
| Dependence |  |  |  |
| 2. Managing money | No difficulty = 0; Some difficulty = 0.33;  Much difficulty = 0.66; Unable to do = 1 | 1999-2000: Data File: PFQ.xpt;  Component: PFQ060a  2001-2002: Data File: PFQ_B.xpt;  Component: PFQ060a  2003-2018: Data File: PFQ_C.xpt~PFQ_J.xpt;  Component: PFQ061a | 11,287 (52.23%) |
| 3. Stooping, crouching, kneeling difficulty | No difficulty = 0; Some difficulty = 0.33;  Much difficulty = 0.66; Unable to do = 1 | 1999-2000: Data File: PFQ.xpt;  Component: PFQ060d  2001-2002: Data File: PFQ_B.xpt;  Component: PFQ060d  2003-2018: Data File: PFQ_C.xpt~PFQ_J.xpt;  Component: PFQ061d | 11,062 (51.19%) |
| 4. Lifting or carrying difficulty | No difficulty = 0; Some difficulty = 0.33;  Much difficulty = 0.66; Unable to do = 1 | 1999-2000: Data File: PFQ.xpt;  Component: PFQ060e  2001-2002: Data File: PFQ_B.xpt;  Component: PFQ060e  2003-2018: Data File: PFQ_C.xpt~PFQ_J.xpt;  Component: PFQ061e | 11,079 (51.27%) |
| 5. House chore difficulty | No difficulty = 0; Some difficulty = 0.33;  Much difficulty = 0.66; Unable to do = 1 | 1999-2000: Data File: PFQ.xpt;  Component: PFQ060f  2001-2002: Data File: PFQ_B.xpt;  Component: PFQ060f  2003-2018: Data File: PFQ_C.xpt~PFQ_J.xpt;  Component: PFQ061f | 11,209 (51.87%) |
| 6. Preparing meals difficulty | No difficulty = 0; Some difficulty = 0.33;  Much difficulty = 0.66; Unable to do = 1 | 1999-2000: Data File: PFQ.xpt;  Component: PFQ060g  2001-2002: Data File: PFQ_B.xpt;  Component: PFQ060g  2003-2018: Data File: PFQ_C.xpt~PFQ_J.xpt;  Component: PFQ061g | 11,289 (52.24%) |
| 7. Standing up from armless chair difficulty | No difficulty = 0; Some difficulty = 0.33;  Much difficulty = 0.66; Unable to do = 1 | 1999-2000: Data File: PFQ.xpt;  Component: PFQ060i  2001-2002: Data File: PFQ_B.xpt;  Component: PFQ060i  2003-2018: Data File: PFQ_C.xpt~PFQ_J.xpt;  Component: PFQ061i | 10,989 (50.85%) |
| 8. Getting in and out of bed difficulty | No difficulty = 0; Some difficulty = 0.33;  Much difficulty = 0.66; Unable to do = 1 | 1999-2000: Data File: PFQ.xpt;  Component: PFQ060j  2001-2002: Data File: PFQ_B.xpt;  Component: PFQ060j  2003-2018: Data File: PFQ_C.xpt~PFQ_J.xpt;  Component: PFQ061j | 10,993 (50.87%) |
| 9. Using fork, knife, drinking from cup difficulty | No difficulty = 0; Some difficulty = 0.33;  Much difficulty = 0.66; Unable to do = 1 | 1999-2000: Data File: PFQ.xpt;  Component: PFQ060k  2001-2002: Data File: PFQ_B.xpt;  Component: PFQ060k  2003-2018: Data File: PFQ_C.xpt~PFQ_J.xpt;  Component: PFQ061k | 10,986 (50.84%) |
| 10. Dressing yourself difficulty | No difficulty = 0; Some difficulty = 0.33;  Much difficulty = 0.66; Unable to do = 1 | 1999-2000: Data File: PFQ.xpt;  Component: PFQ060l  2001-2002: Data File: PFQ_B.xpt;  Component: PFQ060l  2003-2018: Data File: PFQ_C.xpt~PFQ_J.xpt;  Component: PFQ061l | 10,990 (50.86%) |
| 11. Standing for long periods difficulty | No difficulty = 0; Some difficulty = 0.33;  Much difficulty = 0.66; Unable to do = 1 | 1999-2000: Data File: PFQ.xpt;  Component: PFQ060m  2001-2002: Data File: PFQ_B.xpt;  Component: PFQ060m  2003-2018: Data File: PFQ_C.xpt~PFQ_J.xpt;  Component: PFQ061m | 11,131 (51.51%) |
| 12. Grasp/holding small objects difficulty | No difficulty = 0; Some difficulty = 0.33;  Much difficulty = 0.66; Unable to do = 1 | 1999-2000: Data File: PFQ.xpt;  Component: PFQ060p  2001-2002: Data File: PFQ_B.xpt;  Component: PFQ060p  2003-2018: Data File: PFQ_C.xpt~PFQ_J.xpt;  Component: PFQ061p | 10,994 (50.88%) |
| 13. Attending social event difficulty | No difficulty = 0; Some difficulty = 0.33;  Much difficulty = 0.66; Unable to do = 1 | 1999-2000: Data File: PFQ.xpt;  Component: PFQ060r  2001-2002: Data File: PFQ_B.xpt;  Component: PFQ060r  2003-2018: Data File: PFQ_C.xpt~PFQ_J.xpt;  Component: PFQ061r | 11,307 (52.33%) |
| 14. Push or pull large objects difficulty | No difficulty = 0; Some difficulty = 0.33;  Much difficulty = 0.66; Unable to do = 1 | 2003-2016: Data File: PFQ_C.xpt~PFQ_I.xpt;  Component: PFQ061t | 13,082 (60.54%) |
| 15. Walking for a quarter mile difficulty | No difficulty = 0; Some difficulty = 0.33;  Much difficulty = 0.66; Unable to do = 1 | 1999-2000: Data File: PFQ.xpt;  Component: PFQ060b  2001-2002: Data File: PFQ_B.xpt;  Component: PFQ060b  2003-2018: Data File: PFQ_C.xpt~PFQ_J.xpt;  Component: PFQ061b | 12,435 (57.55%) |
| 16. Walking up 10 steps difficulty | No difficulty = 0; Some difficulty = 0.33;  Much difficulty = 0.66; Unable to do = 1 | 1999-2000: Data File: PFQ.xpt;  Component: PFQ060c  2001-2002: Data File: PFQ_B.xpt;  Component: PFQ060c  2003-2018: Data File: PFQ_C.xpt~PFQ_J.xpt;  Component: PFQ061c | 12,411 (57.43%) |
| 1. Leisure activity at home difficulty | No difficulty = 0; Some difficulty = 0.33;  Much difficulty = 0.66; Unable to do = 1 | 1999-2000: Data File: PFQ.xpt;  Component: PFQ060s  2001-2002: Data File: PFQ_B.xpt;  Component: PFQ060s  2003-2018: Data File: PFQ_C.xpt~PFQ_J.xpt;  Component: PFQ061s | 11,001 (50.91%) |
| Depressive Symptoms |  |  |  |
| 18. Have little interest in doing things | 1999-2004: Every day, nearly every day = 1, Most days = 0.75, about half the days = 0.50, less than half the days = 0.25, Not at all =0;  2005-2018: Nearly every day = 1, More than half the days = 0.66, Several days = 0.33, Not at all = 0 | 1999-2000: Data File: CIQMDEP.xpt;  Component: CIQD008, CIQD009  2001-2002: Data File: CIQMDEP_B.xpt;  Component: CIQD008, CIQD009  2003-2004: Data File: CIQMDEP_C.xpt;  Component: CIQD008, CIQD009  2005-2018: Data File: DPQ_D.xpt~DPQ_I.xpt;  Component: DPQ010 | 5,751 (26.61%) |
| 19. Feeling down, depressed, or hopeless | 1999-2004: Every day, nearly every day = 1, Most days = 0.75, about half the days = 0.50, less than half the days = 0.25, Not at all =0;  2005-2018: Nearly every day = 1, More than half the days = 0.66, Several days = 0.33, Not at all = 0 | 1999-2000: Data File: CIQMDEP.xpt;  Component: CIQD001, CIQD002  2001-2002: Data File: CIQMDEP_B.xpt;  Component: CIQD001, CIQD002  2003-2004: Data File: CIQMDEP_C.xpt;  Component: CIQD001, CIQD002  2005-2018: Data File: DPQ_D.xpt~DPQ_I.xpt;  Component: DPQ020 | 5,671 (26.24%) |
| 20. Trouble sleeping or sleeping too much | 1999-2004: Every night = 1, Nearly every night = 0.66, less often = 0.33, Not at all = 0  2005-2018: Nearly every day = 1, More than half the days = 0.66, Several days = 0.33, Not at all = 0 | 1999-2000: Data File: CIQMDEP.xpt;  Component: CIQD025, CIQD026  2001-2002: Data File: CIQMDEP_B.xpt;  Component: CIQD025, CIQD026  2003-2004: Data File: CIQMDEP_C.xpt;  Component: CIQD025, CIQD026  2005-2018: Data File: DPQ_D.xpt~DPQ_I.xpt;  Component: DPQ030 | 6,308 (29.19%) |
| 21. Feeling tired or having little energy | Nearly every day = 1, More than half the days = 0.66, Several days = 0.33, Not at all = 0 | 2005-2018: Data File: DPQ_D.xpt~DPQ_I.xpt;  Component: DPQ040 | 6,413 (29.68%) |
| 22. Poor appetite or overeating | 1999-2004: Yes = 1, No = 0  2005:2018: Nearly every day = 1, More than half the days = 0.66, Several days = 0.33, Not at all = 0 | 1999-2000: Data File: CIQMDEP.xpt;  Component: CIQD019, CIQD022  2001-2002: Data File: CIQMDEP_B.xpt;  Component: CIQD019, CIQD022  2003-2004: Data File: CIQMDEP_C.xpt;  Component: CIQD019, CIQD022  2005-2018: Data File: DPQ_D.xpt~DPQ_I.xpt;  Component: DPQ050 | 6,305 (29.18%) |
| 23. Feeling bad about yourself | 1999-2004: Yes = 1, No = 0  2005:2018: Nearly every day = 1, More than half the days = 0.66, Several days = 0.33, Not at all = 0 | 1999-2000: Data File: CIQMDEP.xpt;  Component: CIQD029  2001-2002: Data File: CIQMDEP_B.xpt;  Component: CIQD029  2003-2004: Data File: CIQMDEP_C.xpt;  Component: CIQD029  2005-2018: Data File: DPQ_D.xpt~DPQ_I.xpt;  Component: DPQ060 | 6,312 (29.21%) |
| 24.Trouble concentrating on things | 1999-2004: Yes = 1, No = 0  2005:2018: Nearly every day = 1, More than half the days = 0.66, Several days = 0.33, Not at all = 0 | 1999-2000: Data File: CIQMDEP.xpt;  Component: CIQD043  2001-2002: Data File: CIQMDEP_B.xpt;  Component: CIQD043  2003-2004: Data File: CIQMDEP_C.xpt;  Component: CIQD043  2005-2018: Data File: DPQ_D.xpt~DPQ_I.xpt;  Component: DPQ070 | 6,309 (29.20%) |
| Comorbidities |  |  |  |
| 25. Arthritis | Yes = 1, No = 0 | Data File: MCQ.xpt~MCQ_J.xpt  Component: MCQ160A | 32 (0.15%) |
| 26. Thyroid problems | Yes = 1, No = 0 | 1999-2000: Data File: MCQ.xpt;  Component: MCQ160I  2001-2002: Data File: MCQ_B.xpt;  Component: MCD160M  2003-2018: Data File: MCQ_C~MCQ_J.xpt;  Component: MCQ160M | 33 (0.15%) |
| 27. Chronic bronchitis | Yes = 1, No = 0 | Data File: MCQ.xpt~MCQ_J.xpt  Component: MCQ160K | 43 (0.20%) |
| 28. Cancer | Yes = 1, No = 0 | Data File: MCQ.xpt~MCQ_J.xpt  Component: MCQ220 | 11 (0.05%) |
| 29. Congestive heart failure | Yes = 1, No = 0 | Data File: MCQ.xpt~MCQ_J.xpt  Component: MCQ160B | 34 (0.16%) |
| 30. Coronary heart disease | Yes = 1, No = 0 | Data File: MCQ.xpt~MCQ_J.xpt  Component: MCQ160C | 43 (0.20%) |
| 31. Angina/Angina pectoris | Yes = 1, No = 0 | Data File: MCQ.xpt~MCQ_J.xpt  Component: MCQ160D | 58 (0.27%) |
| 32. Heart attack | Yes = 1, No = 0 | Data File: MCQ.xpt~MCQ_J.xpt  Component: MCQ160E | 18 (0.08%) |
| 33. Stroke | Yes = 1, No = 0 | Data File: MCQ.xpt~MCQ_J.xpt  Component: MCQ160F | 3 (0.01%) |
| 34. Hypertension | Yes = 1, No = 0 | Data File: BPQ.xpt~BPQ_J.xpt  Component: BPQ020 | 58 (0.27%) |
| 35. Diabetes | Yes = 1, No = 0 | Data File: DIQ.xpt~DIQ_J.xpt  Component: DIQ010 | 4 (0.02%) |
| 36. Weak/failing kidneys | Yes = 1, No = 0 | 1999-2000: Data File: KIQ.xpt;  Component: KIQ020  2001-2018: Data File: KIQ_U_B.xpt~KIQ_U_J.xpt;  Component: KIQ022 | 29 (0.13%) |
| 37. Urinary Leakage | 1999-2000: Yes = 1, No = 0  2001-2018: Greatly = 1, Very much = 0.75, Somewhat = 0.5, Only a little = 0.25 | 1999-2000: Data File: KIQ.xpt;  Component: KIQ040  2001-2018: Data File: KIQ_U_B.xpt~KIQ_U_J.xpt;  Component: KIQ050 | 13,847 (64.08%) |
| Hospital Utilization and Access to Care |  |  |  |
| 38. Self-rated health | Fair, poor = 1, Excellent, Very good, good = 0 | Data File: HUQ.xpt~HUQ_J.xpt  Component: HUQ010 | No missing value |
| 39. Health now compared with 1 year ago | Worse = 1, About the same, better = 0 | Data File: HUQ.xpt~HUQ_J.xpt  Component: HUQ020 | No missing value |
| 40. Overnight hospital patient in past year | Yes = 1, No = 0 | 1999-2000: Data File: HUQ.xpt;  Component: HUQ070  2001-2002: Data File: HUQ_B.xpt;  Component: HUD070  2003-2018: Data File: HUQ_C~HUQ_J.xpt;  Component: HUQ071 | 8 (0.04%) |
| 41. Frequency of health care use during past year | None = 0, 1-4 = 0.5, 5 and more = 1 | 1999-2012: Data File: HUQ.xpt~HUQ_G.xpt;  Component: HUQ050  2013-2018: Data File: HUQ_H.xpt~HUQ_J.xpt;  Component: HUQ051 | 9 (0.04%) |
| 42. Number of prescribed medications | None = 0, 1-4 = 0.5, 5 and more = 1 | 1999-2002: Data File: RXQ_RX.xpt~RXQ_RX_B.xpt;  Component: RXD030, RXD295  2013-2018: Data File: RXQ_RX_C.xpt~RXQ_RX_J.xpt;  Component: RXDUSE, RXDCOUNT | No missing value |
| Physical Anthropometry |  |  |  |
| 43. Body mass index | < 18.5, ≥30 = 1  25-30 = 0.5  18.5-25 = 0 | Data File: BMX.xpt~BMX_J.xpt  Component: BMXBMI | No missing value |
| Laboratory Values |  |  |  |
| 44. Glycohemoglobin (%) | 0%-5.7% = 0, >5.7% = 1 | 1999-2000: Data File: LAB10.xpt;  Component: LBXGH  2001-2004: Data File: L10_B~L10_C.xpt;  Component: LBXGH  2005-2018: Data File: GHB_D~GHB_J.xpt;  Component: LBXGH | 36 (0.17%) |
| 45. Red blood cell count (million cells/mL) | MALE: 4.7-6.1 = 0, Other = 1 FEMALE: 4.2-5.4 = 0, Other = 1 | 1999-2000: Data File: LAB25.xpt;  Component: LBXRBCSI  2001-2004: Data File: L25_B~L25_C.xpt;  Component: LBXRBCSI  2005-2018: Data File: CBC_D~CBC_J.xpt;  Component: LBXRBCSI | 41 (0.19%) |
| 46. Hemoglobin (g/dL) | MALE: 13.5-18 = 0, Other = 1 FEMALE: 12-16 = 0, Other = 1 | 1999-2000: Data File: LAB25.xpt;  Component: LBXHGB  2001-2004: Data File: L25_B~L25_C.xpt;  Component: LBXHGB  2005-2018: Data File: CBC_D~CBC_J.xpt;  Component: LBXHGB | 41 (0.19%) |
| 47. Red cell distribution width (%) | 11.6-14.6 = 0, Other = 1 | 1999-2000: Data File: LAB25.xpt;  Component: LBXRDW  2001-2004: Data File: L25_B~L25_C.xpt;  Component: LBXRDW  2005-2018: Data File: CBC_D~CBC_J.xpt;  Component: LBXRDW | 41 (0.19%) |
| 48. Lymphocyte percent (%) | 20-40 = 0, Other = 1 | 1999-2000: Data File: LAB25.xpt;  Component: LBXLYPCT  2001-2004: Data File: L25_B~L25_C.xpt;  Component: LBXLYPCT  2005-2018: Data File: CBC_D~CBC_J.xpt;  Component: LBXLYPCT | 80 (0.37%) |
| 49. Segmented neutrophils percent (%) | 40-80 = 0, Other = 1 | 1999-2000: Data File: LAB25.xpt;  Component: LBXNEPCT  2001-2004: Data File: L25_B~L25_C.xpt;  Component: LBXNEPCT  2005-2018: Data File: CBC_D~CBC_J.xpt;  Component: LBXNEPCT | 80 (0.37%) |

For participants with missing data on certain frailty-related items, the frailty score was calculated by dividing the total score of available frailty-related items by the total number of items for which the participant had data. This method was consistent with the procedure described by Hakeem et al. (Reference: Hakeem, F. F., Bernabé, E., & Sabbah, W. (2021). Association Between Oral Health and Frailty Among American Older Adults. Journal of the American Medical Directors Association, 22(3), 559–563.e2. https://doi.org/10.1016/j.jamda.2020.07.023).

Supplementary Table S9. Proportions of missing value

| Characteristics | Overall participants (n = 21609) | |
| --- | --- | --- |
|  | N | Percentage, % |
| Poverty income ratio | 1638/21609 | 7.58 |
| Education | 12/21609 | 0.56 |
| Smoking status | 13/21609 | 0.60 |
| Waist circumference | 234/21609 | 1.08 |
| Systolic blood pressure | 64/21609 | 0.30 |
| Estimated glomerular filtration rate | 11/21609 | 0.05 |
| Physical activity | 6/21609 | 0.03 |
| Hemoglobin A1c | 36/21609 | 0.17 |
| 10-year cardiovascular disease risk score | 77/21609 | 0.36 |
| Total cholesterol | 6/21609 | 0.03 |
| High-density lipoprotein cholesterol | 7/21609 | 0.03 |
| Antihypertension medication | 12/21609 | 0.06 |
| Statin | 12/21609 | 0.06 |
| Systemic immune inflammation index | 80/21609 | 0.37 |
| Healthy Eating Index-2020 | 3/21609 | 0.01 |
| Life’s Simple 7 | 920/21609 | 4.26 |

Supplementary Table S10. Knots selection of restricted cubic spline analysis

| Outcome | Number of knots | Akaike information criterion | Bayesian information criterion | Final selected number of knots |
| --- | --- | --- | --- | --- |
| All-cause mortality | 3 | 52387.0 | 52495.8 | 3 |
|  | 4 | 52387.3 | 52502.2 |  |
|  | 5 | 52389.5 | 52510.5 |  |
|  | 6 | 52391.4 | 52518.4 |  |
|  |  |  |  |  |
| Cardiovascular mortality | 3 | 12843.1 | 12927.1 | 3 |
|  | 4 | 12842.7 | 12931.5 |  |
|  | 5 | 12844.6 | 12937.9 |  |
|  | 6 | 12843.6 | 12941.7 |  |
|  |  |  |  |  |
| Non-cardiovascular mortality | 3 | 39514.0 | 39617.6 | 3 |
|  | 4 | 39515.6 | 39624.9 |  |
|  | 5 | 39517.4 | 39632.5 |  |
|  | 6 | 39519.2 | 39640.0 |  |

Supplementary Table S11. Baseline characteristics of all participants categorized by CKM staging

| Characteristics | All  N = 21609 | Non-CKM  N = 1290 | CKM Stage 1  N = 2506 | CKM Stage 2  N = 13478 | CKM Stage 3  N = 2011 | CKM Stage 4  N = 2324 |
| --- | --- | --- | --- | --- | --- | --- |
| Age, years | 52.0 (38.0, 65.0) | 32.0 (25.0, 43.0) | 39.0 (30.0, 51.0) | 49.0 (37.0, 61.0) | 77.0 (72.0, 80.0) | 68.0 (60.0, 76.0) |
| Male, n (%) | 11741 (54.3) | 517 (37.5) | 1257 (51.7) | 7193 (54.2) | 1275 (58.2) | 1499 (62.0) |
| Ethnicity, n (%) |  |  |  |  |  |  |
| Non-Hispanic White | 10694 (49.5) | 734 (78.4) | 1081 (69.2) | 6185 (72.2) | 1277 (82.4) | 1417 (79.8) |
| Non-Hispanic Black | 4296 (19.9) | 188 (6.89) | 489 (9.9) | 2866 (10.2) | 325 (8.1) | 428 (8.9) |
| Mexican American | 3268 (15.1) | 145 (5.0) | 446 (8.8) | 2215 (6.8) | 224 (3.2) | 238 (3.7) |
| Hispanic and Others | 3351 (15.5) | 223 (9.7) | 490 (12.2) | 2212 (10.7) | 185 (6.3) | 241 (7.6) |
| Body mass index, kg/m^2^ | 28.2 (24.6, 32.4) | 22.1 (20.5, 23.5) | 27.2 (25.3, 30.2) | 28.7 (25.2, 33.0) | 28.7 (25.2, 30.1) | 28.3 (25.3, 32.5) |
| Waist circumference, cm | 98.0 (89.0, 109.3) | 78.6 (74.1, 83.2) | 93.9 (87.3, 101.8) | 99.5 (90.2, 109.7) | 101.0 (93.0, 109.3) | 101.8 (93.5, 111.8) |
| Education, n (%) |  |  |  |  |  |  |
| Less than high school | 1945 (9.0) | 46 (2.0) | 177 (3.7) | 1115 (3.6) | 324 (9.1) | 283 (7.0) |
| High school or equivalent | 7794 (36.1) | 337 (23.2) | 762 (27.7) | 4959 (33.9) | 764 (38.0) | 972 (41.7) |
| College or above | 11870 (54.9) | 907 (74.8) | 1567 (68.6) | 7404 (62.5) | 923 (52.9) | 1069 (51.3) |
| Physical activity, n (%)  Less than moderate  Moderate  Vigorous | 10125 (46.9)  6731 (31.1)  4753 (22.0) | 610 (44.5)  333 (26.4)  347 (29.1) | 1188 (44.8)  669 (27.1)  649 (28.1) | 6265 (42.3)  4087 (32.4)  3126 (25.3) | 984 (46.4)  766 (39.6)  261 (14.0) | 1078 (42.2)  876 (39.2)  370 (18.6) |
| Smoking status, n (%)  Never smoker  Former smoker  Current smoker | 11369 (52.6)  6055 (28.0)  4185 (19.4) | 827 (62.3)  198 (16.8)  265 (20.9) | 1516 (58.3)  556 (25.6)  434 (16.1) | 7158 (52.6)  3384 (26.5)  2936 (20.9) | 989 (49.4)  890 (45.3)  132 (5.3) | 879 (36.8)  1027 (43.5)  418 (19.8) |
| Alcohol consumption, n (%)  Non-drinker  Mild to moderate  Heavy | 13098 (60.6)  3113 (14.4)  1749 (8.1) | 639 (47.0)  419 (34.8)  232 (18.2) | 1296 (47.1)  788 (35.5)  422 (17.4) | 7713 (53.6)  3562 (29.3)  2203 (17.1) | 1692 (82.4)  248 (14.2)  71 (3.5) | 1758 (72.9)  381 (18.9)  185 (8.2) |
| Laboratory indicators |  |  |  |  |  |  |
| Hemoglobin A1c, % | 5.5 (5.2, 5.9) | 5.1 (5.0, 5.3) | 5.3 (5.1, 5.6) | 5.5 (5.2, 5.8) | 5.8 (5.4, 6.4) | 5.7 (5.4, 6.2) |
| Total Cholesterol, mg/dL | 195.0 (169.0, 223.0) | 181.0 (157.0, 202.0) | 191.5 (167.0, 214.0) | 203.0 (177, 230.0) | 195.0 (170.0, 220.0) | 183.0 (157.0, 215.0) |
| HDL-C, mg/dL | 51.0 (42.0, 62.0) | 61.0 (53.0, 71.0) | 56.0 (50.0, 65.0) | 48.0 (40.0, 59.0) | 48.0 (40.0, 59.0) | 46.0 (38.0, 57.0) |
| eGFR, ml/min/1.73m^2^ | 92.6 (76.8, 107.4) | 105.5 (92.6, 117.4) | 102.3 (89.3, 114.2) | 94.6 (81.4, 107.9) | 64.4 (51.5, 80.3) | 73.9 (57.7, 88.1) |
| UACR, mg/g | 7.0 (4.5, 14.3) | 5.3 (3.8, 8.1) | 4.9 (3.5, 7.3) | 6.8 (4.4, 13.3) | 13.2 (6.8, 38.3) | 10.0 (5.6, 30.6) |
| Multidimensional score |  |  |  |  |  |  |
| Oxidative Balance Score | 21.0 (15.0, 26.0) | 24.0 (17.0, 29.0) | 22.0 (16.0, 27.0) | 20.0 (14.0, 26.0) | 19.0 (13.0, 24.0) | 19.0 (13.0, 24.0) |
| 10-year CVD risk score | 4.3 (1.2, 12.7) | 0.5 (0.2, 1.2) | 1.0 (0.4, 3.0) | 3.8 (1.3, 8.9) | 25.6 (22.5, 30.2) | 17.1 (8.6, 26.1) |
| SII | 468.0 (334.4, 659.0) | 410.4 (302.3, 590.3) | 433.7 (303.5, 591.4) | 477.7 (345.6, 661.4) | 525.0 (356.5, 739.2) | 505.9 (356.9, 741.0) |
| Life’s Simple 7 score | 8.0 (7.0, 10.0) | 11.11 (0.06) | 9.55 (0.04) | 7.95 (0.03) | 7.43 (0.05) | 7.17 (0.05) |
| Frailty score×10 | 1.2 (0.8, 1.9) | 0.8 (0.4, 1.2) | 0.8 (0.6, 1.3) | 1.2 (0.8, 1.7) | 1.6 (1.1, 2.2) | 2.3 (1.6, 3.0) |
| HEI | 50.9 (41.6, 60.9) | 51.9 (42.6, 61.8) | 51.1 (41.9, 61.7) | 50.5 (41.4, 60.1) | 55.0 (46.1, 64.8) | 52.2 (42.7, 62.3) |

Abbreviations: CKM, cardiovascular-kidney-metabolic syndrome; CVD, cardiovascular disease; DBP, diastolic blood pressure; eGFR, estimated glomerular filtration rate; HDL-C, high-density lipoprotein cholesterol; SBP, systolic blood pressure; SII, systemic immune-inflammation index; UACR, urinary albumin to creatinine ratio.

Supplementary Table S12. Subgroup analysis of exploring the interaction between oxidative balance score and mortality outcomes

|  | All cause mortality | | | Cardiovascular mortality | | | Non-cardiovascualr mortality | | |
| --- | --- | --- | --- | --- | --- | --- | --- | --- | --- |
|  | HR (95% CI) | *P* | *P-interaction* | HR (95% CI) | *P* | *P-interaction* | HR (95% CI) | *P* | *P-interaction* |
| Age ≥65 years |  |  |  |  |  |  |  |  |  |
| Q1 | 1.29 (1.13, 1.46) | < 0.001 | 0.070 | 1.36 (1.07, 1.74) | 0.014 | 0.003 | 1.26 (1.09, 1.47) | 0.002 | 0.369 |
| Q2 | 1.20 (1.05, 1.36) | 0.008 |  | 1.24 (0.96, 1.61) | 0.093 |  | 1.18 (1.01, 1.37) | 0.035 |  |
| Q3 | 1.15 (1.01, 1.30) | 0.032 |  | 1.32 (1.03, 1.68) | 0.026 |  | 1.09 (0.94, 1.27) | 0.249 |  |
| Q4 | *Reference* |  |  | *Reference* |  |  | *Reference* |  |  |
| Age <65 years |  |  |  |  |  |  |  |  |  |
| Q1 | 1.48 (1.21, 1.81) | < 0.001 |  | 2.04 (1.27, 3.29) | 0.003 |  | 1.37 (1.10, 1.72) | 0.006 |  |
| Q2 | 1.51 (1.22, 1.85) | < 0.001 |  | 2.13 (1.31, 3.47) | 0.002 |  | 1.38 (1.10, 1.74) | 0.006 |  |
| Q3 | 1.28 (1.04, 1.57) | 0.020 |  | 1.10 (0.65, 1.86) | 0.725 |  | 1.31 (1.05, 1.64) | 0.017 |  |
| Q4 | *Reference* |  |  | *Reference* |  |  | *Reference* |  |  |
|  |  |  |  |  |  |  |  |  |  |
| Male |  |  |  |  |  |  |  |  |  |
| Q1 | 1.35 (1.18, 1.55) | < 0.001 | 0.824 | 1.51 (1.16, 1.97) | 0.002 | 0.441 | 1.30 (1.10, 1.53) | 0.002 | 0.618 |
| Q2 | 1.28 (1.11, 1.47) | < 0.001 |  | 1.31 (0.99, 1.73) | 0.060 |  | 1.27 (1.07, 1.50) | 0.005 |  |
| Q3 | 1.24 (1.07, 1.42) | 0.003 |  | 1.21 (0.92, 1.60) | 0.179 |  | 1.24 (1.06, 1.46) | 0.009 |  |
| Q4 | *Reference* |  |  | *Reference* |  |  | *Reference* |  |  |
| Female |  |  |  |  |  |  |  |  |  |
| Q1 | 1.24 (1.04, 1.47) | 0.016 |  | 1.23 (0.84, 1.80) | 0.290 |  | 1.24 (1.02, 1.50) | 0.031 |  |
| Q2 | 1.26 (1.06, 1.50) | 0.009 |  | 1.54 (1.06, 2.22) | 0.023 |  | 1.19 (0.98, 1.45) | 0.086 |  |
| Q3 | 1.11 (0.94, 1.31) | 0.229 |  | 1.32 (0.92, 1.90) | 0.128 |  | 1.05 (0.87, 1.27) | 0.600 |  |
| Q4 | *Reference* |  |  | *Reference* |  |  | *Reference* |  |  |
|  |  |  |  |  |  |  |  |  |  |
| BMI ≥30 kg/m^2^ | |  |  |  |  |  |  |  |  |
| Q1 | 1.23 (1.00, 1.50) | 0.047 | 0.974 | 1.37 (0.92, 2.03) | 0.122 | 0.976 | 1.29 (1.89, 1.10) | 0.820 | 0.958 |
| Q2 | 1.23 (1.00, 1.52) | 0.049 |  | 1.29 (0.85, 1.905) | 0.236 |  | 1.29 (1.89, 1.10) | 0.820 |  |
| Q3 | 1.13 (0.92, 1.39) | 0.256 |  | 1.15 (0.76, 1.75) | 0.502 |  | 0.88 (0.78, 0.99) | 0.038 |  |
| Q4 | *Reference* |  |  | *Reference* |  |  | *Reference* |  |  |
| BMI <30 kg/m^2^ | |  |  |  |  |  |  |  |  |
| Q1 | 1.34 (1.18, 1.52) | < 0.001 |  | 1.40 (1.08, 1.82) | 0.012 |  | 1.32 (1.14, 1.53) | < 0.001 |  |
| Q2 | 1.28 (1.12, 1.45) | < 0.001 |  | 1.39 (1.06, 1.82) | 0.016 |  | 1.24 (1.07, 1.44) | 0.005 |  |
| Q3 | 1.20 (1.05, 1.36) | 0.005 |  | 1.28 (0.99, 1.66) | 0.063 |  | 1.17 (1.01, 1.35) | 0.033 |  |
| Q4 | *Reference* |  |  | *Reference* |  |  | *Reference* |  |  |
|  |  |  |  |  |  |  |  |  |  |
| CKM-stage 1/2 |  |  |  |  |  |  |  |  |  |
| Q1 | 1.33 (1.13, 1.58) | < 0.001 | 0.522 | 1.34 (0.91, 1.98) | 0.142 | 0.710 | 1.33 (1.10, 1.60) | 0.003 | 0.569 |
| Q2 | 1.31 (1.10, 1.56) | 0.002 |  | 1.35 (0.90, 2.02) | 0.142 |  | 1.30 (1.07, 1.58) | 0.007 |  |
| Q3 | 1.18 (1.00, 1.40) | 0.051 |  | 1.11 (0.75, 1.66) | 0.592 |  | 1.20 (0.99, 1.44) | 0.058 |  |
| Q4 | *Reference* |  |  | *Reference* |  |  | *Reference* |  |  |
| CKM-stage 3/4 |  |  |  |  |  |  |  |  |  |
| Q1 | 1.26 (1.09, 1.44) | 0.001 |  | 1.45 (1.11, 1.88) | 0.006 |  | 1.19 (1.10, 1.40) | 0.038 |  |
| Q2 | 1.20 (1.04, 1.39) | 0.011 |  | 1.38 (1.05, 1.81) | 0.019 |  | 1.14 (0.96, 1.35) | 0.127 |  |
| Q3 | 1.16 (1.01, 1.33) | 0.039 |  | 1.32 (1.02, 1.72) | 0.038 |  | 1.10 (0.93, 1.30) | 0.256 |  |
| Q4 | *Reference* |  |  | *Reference* |  |  | *Reference* |  |  |

Models were adjusted for age, sex, race and ethnicity, education level, poverty income ratio, smoking status, alcohol consumption, physical activity.

Abbreviations: BMI, body mass index; CKM, cardiovascular-kidney-metabolic syndrome; CVD, cardiovascular disease.

Supplementary Table S13. Sensitivity analysis of OBS and mortality outcomes in CKM patients after excluding patients died within the first two-year follow-up

|  | All-cause mortality | | Cardiovascular mortality | | Non-cardiovascular mortality | |
| --- | --- | --- | --- | --- | --- | --- |
| OBS quartiles | HR (95% CI) | *P* | HR (95% CI) | *P* | HR (95% CI) | *P* |
| Q1 | 1.29 (1.15, 1.44) | < 0.001 | 1.43 (1.13, 1.80) | 0.002 | 1.25 (1.10, 1.42) | < 0.001 |
| Q2 | 1.26 (1.12, 1.42) | < 0.001 | 1.41 (1.11, 1.78) | 0.004 | 1.22 (1.07, 1.39) | 0.004 |
| Q3 | 1.19 (1.06, 1.33) | 0.003 | 1.31 (1.04, 1.65) | 0.022 | 1.15 (1.01, 1.31) | 0.030 |
| Q4 | *Reference* |  | *Reference* |  | *Reference* |  |

Models were adjusted for age, sex, race and ethnicity, education level, poverty income ratio, smoking status, alcohol consumption, physical activity.

Abbreviations: CI, confidence interval; CKM, cardiovascular-kidney-metabolic syndrome; HR, hazard ratio; OBS, oxidative balance score.

Supplementary Table S14. Sensitivity analysis of OBS and mortality outcomes in CKM patients after excluding patients with cancer

|  | All-cause mortality | | Cardiovascular mortality | | Non-cardiovascular mortality | |
| --- | --- | --- | --- | --- | --- | --- |
| OBS quartiles | HR (95% CI) | *P* | HR (95% CI) | *P* | HR (95% CI) | *P* |
| Q1 | 1.32 (1.17, 1.50) | < 0.001 | 1.47 (1.14, 1.88) | 0.002 | 1.28 (1.11, 1.47) | < 0.001 |
| Q2 | 1.25 (1.10, 1.42) | < 0.001 | 1.41 (1.09, 1.82) | 0.009 | 1.19 (1.03, 1.38) | 0.021 |
| Q3 | 1.22 (1.08, 1.38) | 0.002 | 1.31 (1.02, 1.69) | 0.035 | 1.19 (1.03, 1.37) | 0.018 |
| Q4 | *Reference* |  | *Reference* |  | *Reference* |  |

Models were adjusted for age, sex, race and ethnicity, education level, poverty income ratio, smoking status, alcohol consumption, physical activity.

Abbreviations: CI, confidence interval; CKM, cardiovascular-kidney-metabolic syndrome; HR, hazard ratio; OBS, oxidative balance score.

Supplementary Table S15. Association between OBS and mortality outcomes in CKM patients stratified by optimal risk cut-off points

|  | All-cause mortality |  | Cardiovascular mortality |  | Non-cardiovascular mortality |  |
| --- | --- | --- | --- | --- | --- | --- |
|  | HR (95% CI) | *P* | HR (95% CI) | *P* | HR (95% CI) | *P* |
| OBS <22 | 1.19 (1.11, 1.29) | < 0.001 | 1.27 (1.09, 1.49) | 0.002 | 1.17 (1.07, 1.28) | < 0.001 |
| OBS ≥22 | *Reference* |  | *Reference* |  | *Reference* |  |

Models were adjusted for age, sex, race and ethnicity, education level, poverty income ratio, smoking status, alcohol consumption, physical activity.

Abbreviations: CI, confidence interval; CKM, cardiovascular-kidney-metabolic syndrome; HR, hazard ratio; OBS, oxidative balance score.

Supplementary Table S16. Best hyperparameters of each machine learning model for predicting advanced staging of cardiovascular-kidney-metabolic syndrome in whole population

| Classifiers | Hyperparameters | |
| --- | --- | --- |
| Light gradient boosting machine | n_estimators | 488 |
|  | max_depth | 8 |
|  | learning_rate | 0.01 |
|  | boosting_type | ‘gbdt’ |
|  | objective | ‘binary’ |
|  | num_leaves | 31 |
|  | colsample_bytree | 0.8589388113003151 |
|  | min_child_samples | 59 |
|  | subsample | 0.5228156690739117 |
|  | class_weight | ‘balanced’ |
|  |  |  |
| Random forest | n_estimators | 279 |
|  | max_depth | 11 |
|  | criterion | ‘entropy’ |
|  | min_samples_leaf | 32 |
|  | random_state | 40 |
|  | min_sample_split | 33 |
|  | class_weight | ‘balanced’ |
|  |  |  |
| Logistic regression | C | 10 |
|  | penalty | l2 |
|  | solver | ‘sag’ |
|  | max_iter | 300 |
|  | class_weight | ‘balanced’ |
|  |  |  |
| Support vector machine | C | 1 |
|  | kernel | ‘poly’ |
|  | probability | True |
|  | tol | 0.0001 |
|  | gamma | ‘auto’ |
|  | class_weight | ‘balanced’ |
|  |  |  |
| Multi-layer perceptron | solver | ‘adam’ |
|  | activation | ‘tanh’ |
|  | learning_rate | ‘adaptive’ |
|  | hidden_layer_sizes | (100, 50) |
|  | alpha | 0.01 |
|  | max_iter | 200 |
|  | random_state | 42 |

Supplementary Table S17. Best hyperparameters of each machine learning model for predicting all-cause mortality in patients with cardiovascular-kidney-metabolic syndrome

| Classifiers | Hyperparameters | |
| --- | --- | --- |
| Light gradient boosting machine | n_estimators | 300 |
|  | max_depth | 8 |
|  | learning_rate | 0.02 |
|  | boosting_type | ‘gbdt’ |
|  | objective | ‘binary’ |
|  | num_leaves | 31 |
|  | colsample_bytree | 0.8589388113003151 |
|  | min_child_samples | 59 |
|  | subsample | 0.5228156690739117 |
|  | class_weight | ‘balanced’ |
|  |  |  |
| Random forest | n_estimators | 280 |
|  | max_depth | 18 |
|  | criterion | ‘entropy’ |
|  | min_samples_leaf | 32 |
|  | random_state | 42 |
|  | min_sample_split | 33 |
|  | class_weight | ‘balanced’ |
|  |  |  |
| Logistic regression | C | 10 |
|  | penalty | l2 |
|  | solver | ‘liblinear’ |
|  | max_iter | 300 |
|  | class_weight | ‘balanced’ |
|  |  |  |
| Support vector machine | C | 1 |
|  | kernel | ‘poly’ |
|  | probability | True |
|  | tol | 0.0001 |
|  | gamma | ‘auto’ |
|  | class_weight | ‘balanced’ |
|  |  |  |
| Multi-layer perceptron | solver | ‘adam’ |
|  | activation | ‘tanh’ |
|  | learning_rate | ‘adaptive’ |
|  | hidden_layer_sizes | (100, 50) |
|  | alpha | 0.01 |
|  | max_iter | 300 |
|  | random_state | 42 |

Supplementary Table S18. Performance metrics of each machine learning model for predicting advanced CKM staging in whole population

|  | AUC (95% CI) | Accuracy | Specificity | Precision | Recall | F1-score | G-mean |
| --- | --- | --- | --- | --- | --- | --- | --- |
| Light gradient boosting machine | 0.905 (0.897-0.912) | 0.842 | 0.854 | 0.578 | 0.792 | 0.668 | 0.677 |
| Random forest | 0.902 (0.895-0.909) | 0.847 | 0.867 | 0.592 | 0.769 | 0.669 | 0.675 |
| Logistic regression | 0.811 (0.802-0.821) | 0.669 | 0.632 | 0.358 | 0.818 | 0.498 | 0.541 |
| Support vector machine | 0.885 (0.877-0.893) | 0.845 | 0.869 | 0.590 | 0.749 | 0.660 | 0.665 |
| Multi-layer perceptron | 0.846 (0.837-0.854) | 0.825 | 0.868 | 0.554 | 0.653 | 0.600 | 0.601 |

Abbreviations: AUC, area under curve; CI, confidence interval; CKM, cardiovascular-kidney-metabolic syndrome.

Supplementary Table S19. Performance metrics of each machine learning model for predicting all-cause mortality in CKM patients

|  | AUC (95% CI) | Accuracy | Specificity | Precision | Recall | F1-score | G-mean |
| --- | --- | --- | --- | --- | --- | --- | --- |
| Light gradient boosting machine | 0.875 (0.866-0.883) | 0.815 | 0.827 | 0.441 | 0.753 | 0.556 | 0.576 |
| Random forest | 0.864 (0.856-0.873) | 0.805 | 0.819 | 0.423 | 0.733 | 0.536 | 0.557 |
| Logistic regression | 0.861 (0.853-0.870) | 0.752 | 0.739 | 0.364 | 0.824 | 0.505 | 0.548 |
| Support vector machine | 0.845 (0.836-0.854) | 0.822 | 0.847 | 0.448 | 0.687 | 0.543 | 0.555 |
| Multi-layer perceptron | 0.772 (0.761-0.782) | 0.813 | 0.875 | 0.407 | 0.472 | 0.437 | 0.438 |

Abbreviations: AUC, area under curve; CI, confidence interval; CKM, cardiovascular-kidney-metabolic syndrome.


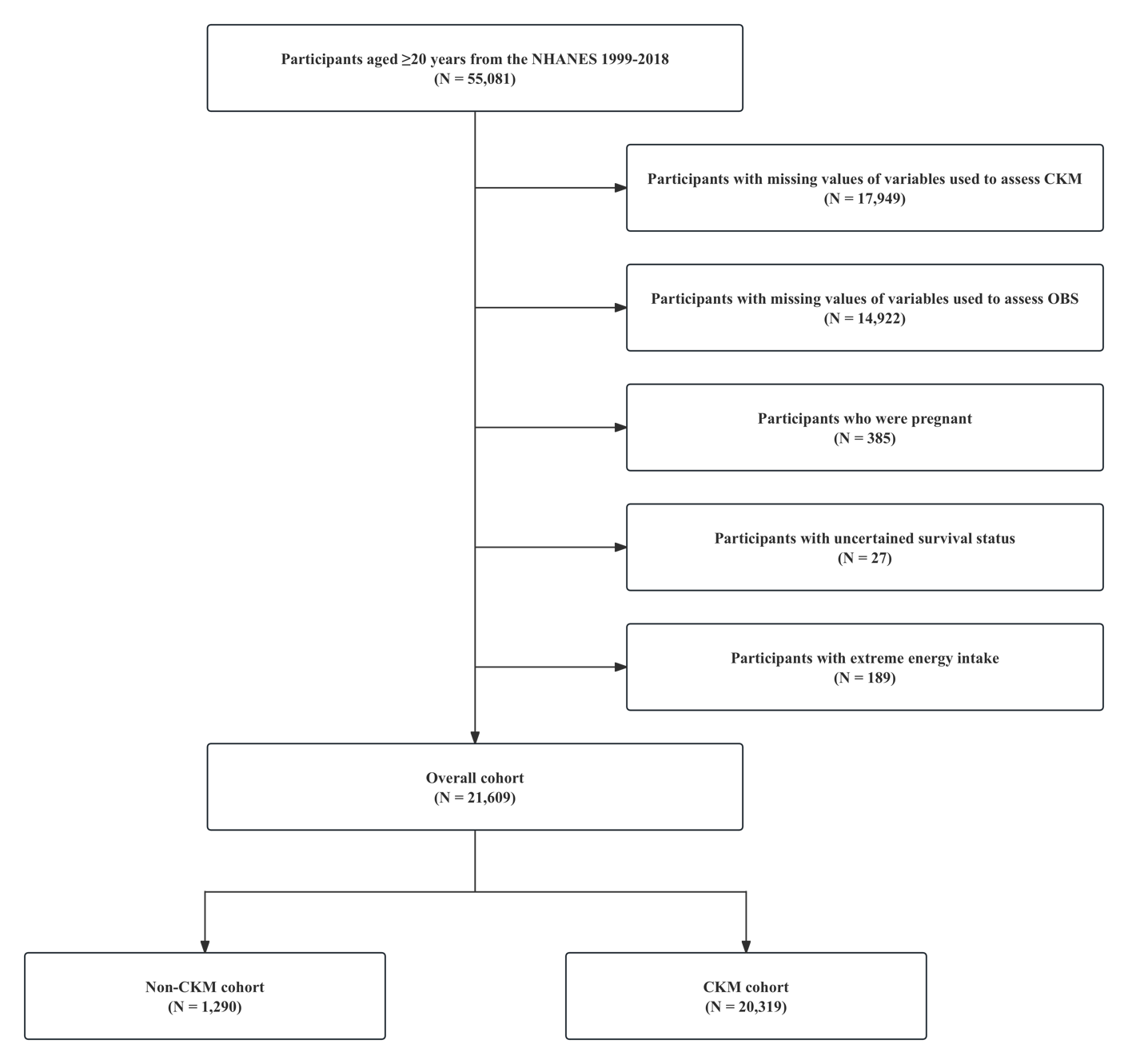


Supplementary Figure S1. Flowchart of this study. CKM, cardiovascular-kidney-metabolic syndrom; NHANES, National Health and Nutrition Examination Survey; OBS, oxidative balance score.

Supplementary Figure S2. Variance inflation factor values for features in the model in predicting advanced staging of cardiovascular-kidney-metabolic syndrome.

Supplementary Figure S3. Variance inflation factor values for features in the model in predicting all-cause mortality of patients with cardiovascular-kidney-metabolic syndrome.


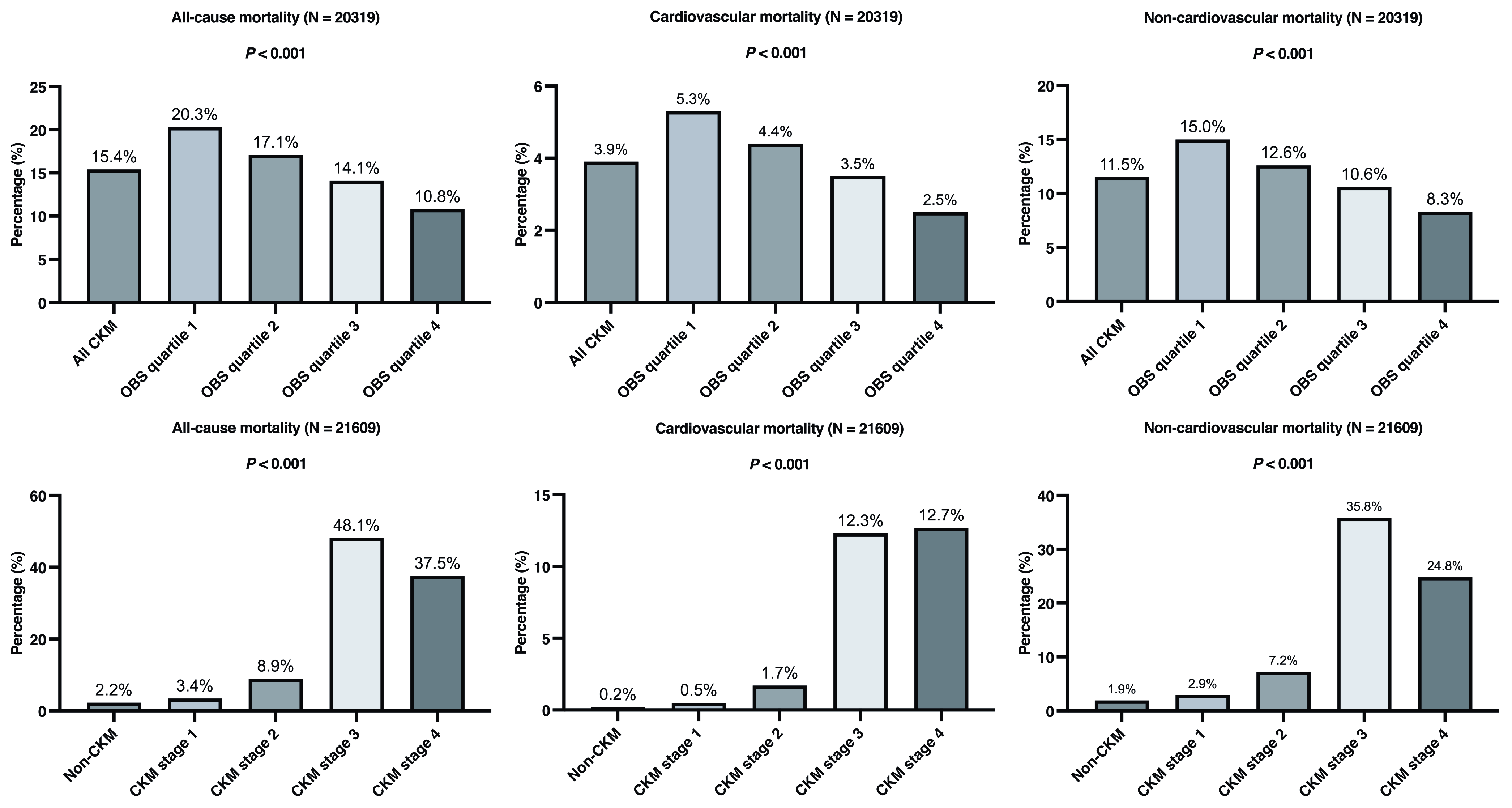


Supplementary Figure S4. Distribution of mortality outcomes by CKM stages and OBS quartiles in CKM patients. P values from the Fisher’s exact test. CKM, cardiovascular-kidney-metabolic syndrome; OBS, oxidative balance score.


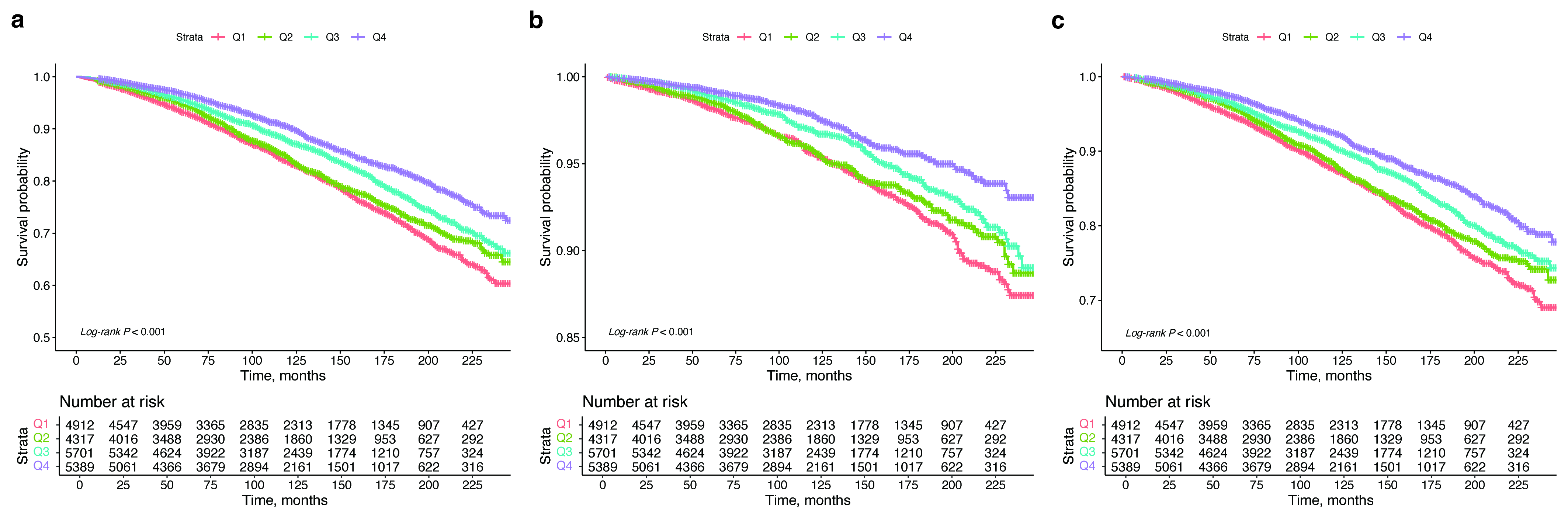


Supplementary Figure S5. Kaplan-Meier survival curves for mortality outcomes across OBS quartiles in CKM patients. (a) All-cause mortality, (b) cardiovascular mortality, (c) non-cardiovascular mortality. Q1: OBS<15; Q2: 15≤ OBS <20; Q3: 20≤ OBS <26; Q4: OBS ≥26. Log-rank P values from log-rank test. CKM, cardiovascular-kidney-metabolic syndrome; OBS, oxidative balance score.


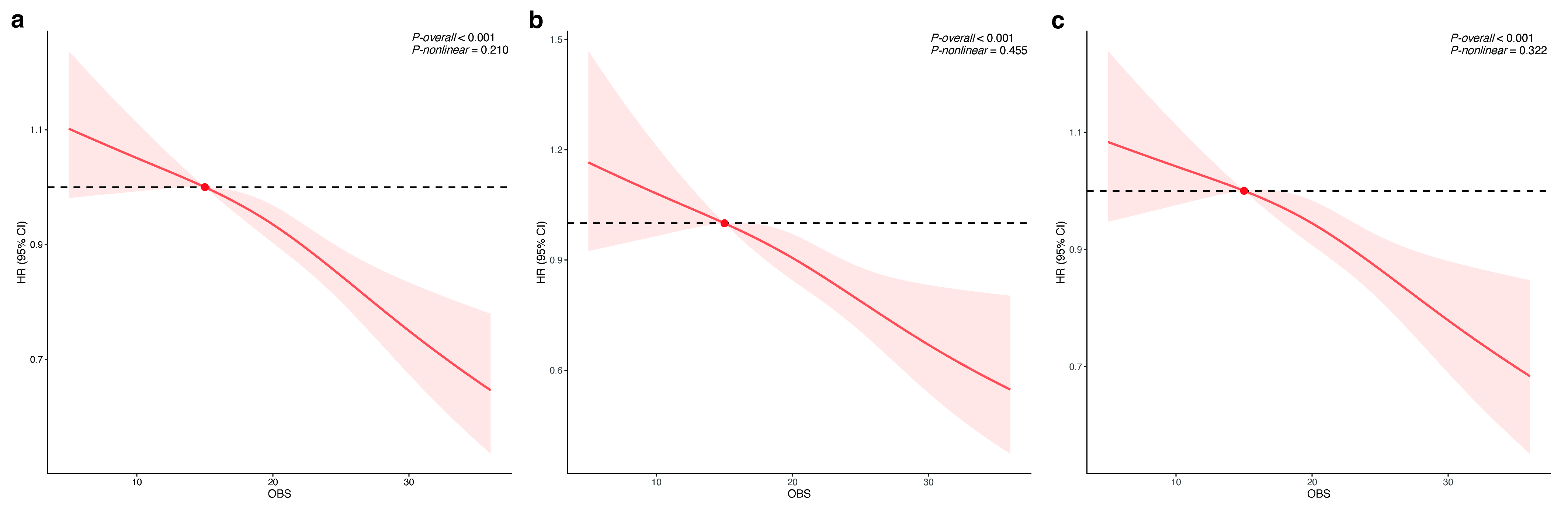


Supplementary Figure S6. Restricted cubic spline analyses for associations between OBS and mortality outcomes in CKM patients. (a) All-cause mortality, (b) cardiovascular mortality, (c) non-cardiovascular mortality. P values from multivariable Cox proportional hazards models adjusted for age, sex, race and ethnicity, education level, poverty income ratio, smoking status, alcohol consumption, physical activity. CI, confidence interval; CKM, cardiovascular-kidney-metabolic syndrome; HR, hazard ratio; OBS, oxidative balance score.

Supplementary Figure S7. Restricted cubic spline analysis of the association between OBS and cardiovascular mortality in patients with cardiovascular-kidney-metabolic syndrom stratified by age (<65 years vs. ≥65 years). P values from multivariable Cox proportional hazards models adjusted for age, sex, race and ethnicity, education level, poverty income ratio, smoking status, alcohol consumption, physical activity. OBS, oxidative balance score.


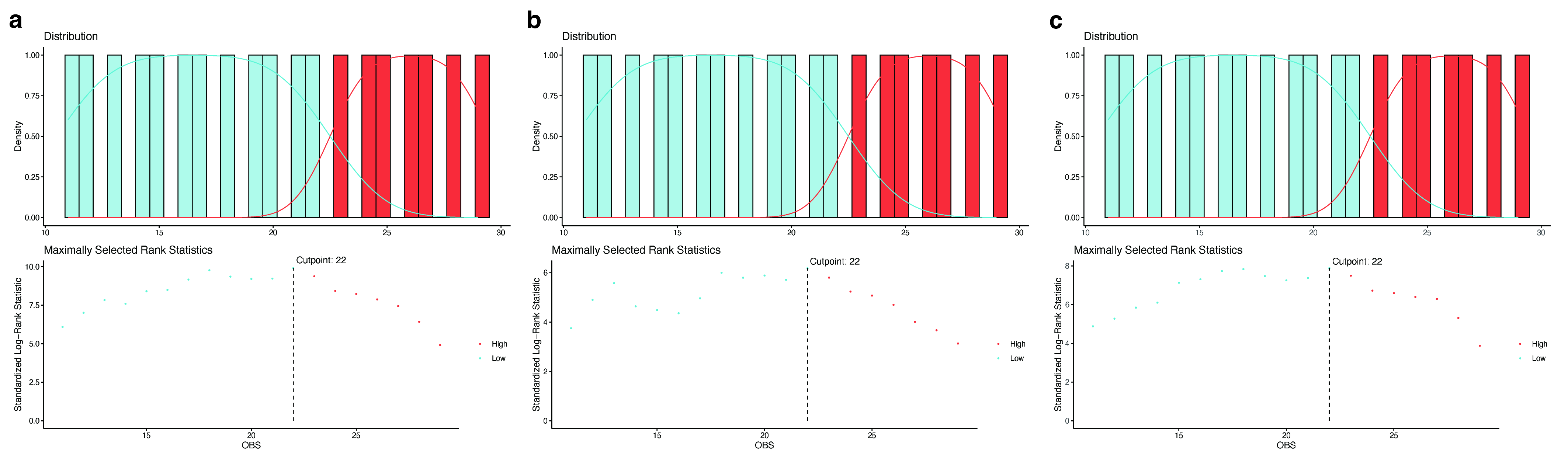


Supplementary Figure S8. Determination of optimal risk stratification cut-off points for OBS on mortality outcomes in CKM patients. (a) All-cause mortality, (b) cardiovascular mortality, (c) non-cardiovascular mortality. CKM, cardiovascular-kidney-metabolic syndrome; OBS, oxidative balance score.
